# Clinical relevance of the transcriptional signature regulated by CDC42 in colorectal cancer

**DOI:** 10.18632/oncotarget.15815

**Published:** 2017-03-01

**Authors:** Fatima Valdés-Mora, Warwick J. Locke, Eva Bandrés, David Gallego-Ortega, Paloma Cejas, Miguel Angel García-Cabezas, Yolanda Colino-Sanguino, Jaime Feliú, Teresa Gómez del Pulgar, Juan Carlos Lacal

**Affiliations:** ^1^ Histone Variants Group, Epigenetics Research Program, Genomics and Epigenetics Division, Garvan Institute of Medical Research, Sydney, New South Wales, Australia; ^2^ St. Vincent's Clinical School, Faculty of Medicine, University of New South Wales Sydney, New South Wales, Australia; ^3^ Epigenetics Research Program, Genomics and Epigenetics Division, Garvan Institute of Medical Research, Sydney, New South Wales, Australia; ^4^ Immunology Unit, Department of Haematology, Complejo Hospitalario de Navarra, Navarra Health Service, Pamplona, Spain; ^5^ Tumour Development Group, The Kinghorn Cancer Centre, Garvan Institute of Medical Research, Sydney, New South Wales, Australia; ^6^ Laboratorio de Oncología Translacional, Servicio de Oncología Médica, IdiPAZ, Madrid, Spain; ^7^ Servicio de Anatomía Patológica, Hospital Universitario La Paz, Madrid, Spain; ^8^ Servicio de Oncología Médica, IdiPAZ, CIBERONC, Madrid, Spain

**Keywords:** colorectal cancer, CACNA2D2, CDC42, tumor suppressor genes, prognostic factor

## Abstract

CDC42 is an oncogenic Rho GTPase overexpressed in colorectal cancer (CRC). Although CDC42 has been shown to regulate gene transcription, the specific molecular mechanisms regulating the oncogenic ability of CDC42 remain unknown. Here, we have characterized the transcriptional networks governed by CDC42 in the CRC SW620 cell line using gene expression analysis. Our results establish that several cancer-related signaling pathways, including cell migration and cell proliferation, are regulated by CDC42. This transcriptional signature was validated in two large cohorts of CRC patients and its clinical relevance was also studied. We demonstrate that three CDC42-regulated genes offered a better prognostic value when combined with CDC42 compared to CDC42 alone. In particular, the concordant overexpression of *CDC42* and silencing of the putative tumor suppressor gene *CACNA2D2* dramatically improved the prognostic value. The *CACNA2D2/CDC42* prognostic classifier was further validated in a third CRC cohort as well as *in vitro* and *in vivo* CRC models. Altogether, we show that CDC42 has an active oncogenic role in CRC via the transcriptional regulation of multiple cancer-related pathways and that CDC42-mediated silencing of *CACNA2D2* is clinically relevant. Our results further support the use of CDC42 specific inhibitors for the treatment of the most aggressive types of CRC.

## INTRODUCTION

Colorectal cancer (CRC) is among the most commonly diagnosed cancers worldwide and one of the leading causes of cancer-related deaths for both males and females [1]. Despite the high resectability rate and a general improvement in therapy, nearly half of all patients with colorectal cancer still die of metastatic disease. This poor outcome underscores the need for new tools to facilitate better management of CRC. The identification of robust prognostic factors able to identify critical events in the development and progression of CRC will be of particularly high value. Therefore, to identify these factors and for an optimal management of the disease, it is essential to elucidate the molecular events involved in the malignant transformation.

CDC42, a member of the Rho family of GTPases, is involved in the regulation of critical cellular functions such as rearrangement of actin cytoskeleton, cell polarity, intracellular trafficking, cell-cycle regulation, cell fate determination and gene transcription [2]. Considering its key role in these diverse cellular processes, it is not surprising that aberrant activation of CDC42 can be oncogenic. To date no activating *CDC42* mutations have been detected in human cancer, but CDC42 has been shown to be overexpressed in many different cancer types such as breast [3, 4], testicular cancer [5], head and neck squamous cell cancer [6], melanoma [7], colorectal cancer [8], and non-small cell lung cancer [9].

CDC42 has been implicated in tumor development and progression through the alteration of its different roles in a tissue-specific manner. We have previously reported the overexpression of CDC42 in human CRC specimens is associated with histopathological grade [8]. The pro-oncogenic role of CDC42 in this tumor type has been further demonstrated in several independent studies, in which the overexpression or silencing of CDC42 either *in vitro* or *in vivo* showed oncogenic-phenotypic effects in different colorectal cancer cell lines and mouse models [10–12]. These studies have demonstrated that the oncogenic impact of CDC42 was due to its well-known regulatory roles in cellular migration [10, 11, 13, 14] and proliferation [13–16]. CDC42 has been demonstrated to regulate the transcription regulation of a specific set of genes, [17] including down-regulation of the tumor suppressor gene ID4 [8]. However, the role of CDC42 in global gene transcriptional regulation in cancer remains poorly understood.

The aim of this work was the identification of new and clinically relevant genes and transcriptional networks regulated by CDC42 in CRC. To this end, we used our previously established cellular models for CDC42 gain or loss of function in the CRC SW620 cell line [8] to profile the transcriptional changes mediated by CDC42. We identified not only transcriptional networks related to functions already described for CDC42 but also novel functions like chromatin regulation or stem-cell-related roles. CDC42 transcriptional signature comprising 57 genes was validated in two CRC cohorts from the The Cancer Genome Atlas (TCGA). We further studied the prognostic significance of this transcriptional signature and found that the combination of *CDC42* expression with *CACNA2D2, LARS2* or *REG1CP* were better prognostic identifiers than each gene alone. Particularly, the most significant combination was found between high *CDC42* and low *CACNA2D2*, this new prognostic classifier was further validated in a third CRC cohort as well as *in vitro* and *in vivo* CRC models. Thus, CDC42 is a useful novel tool as a prognostic factor and a therapeutic target in CRC.

## RESULTS

### Identification of CDC42-driven transcriptional network in SW620 cells

To address if CDC42 regulates oncogenic transcriptional networks in CRC we used our previously generated *in vitro* CRC cellular model [8] to perform gene expression arrays.

**Figure 1:**
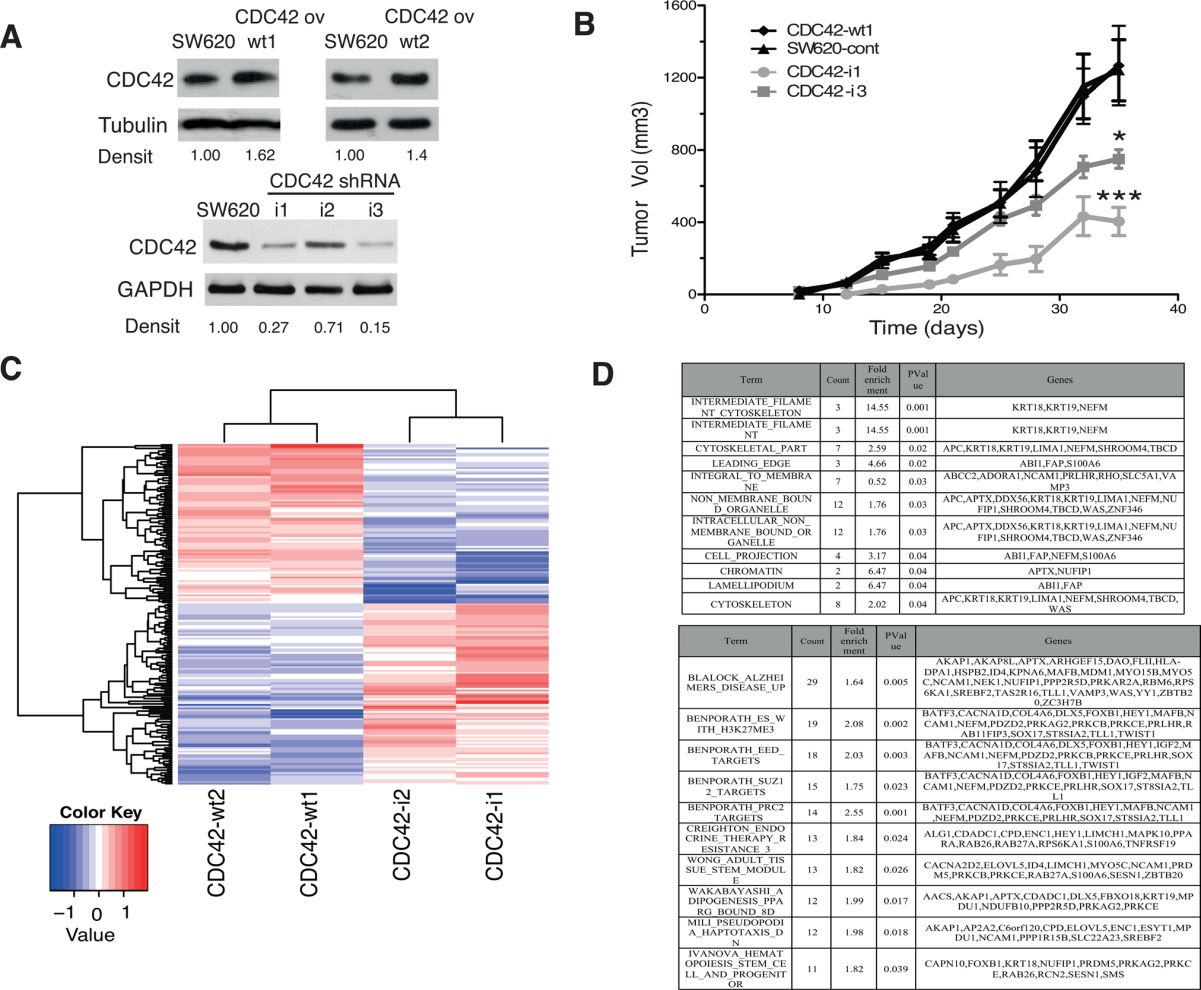
*In vivo* and transcriptional features of SW620 cell clones with altered CDC42 expression (**A**) Protein levels of CDC42 determined by Western blot analysis in the selected SW620 cell clones to perform microarray studies. Tubulin or GAPDH were used as loading controls. Densitometrical analysis (Densit) of relative amount of CDC42 in each cell line compared to expression in parental cell line that was set to 1.0 is shown. Please note that the Western blot for CDC42 ov was cropped from a Western blot containing other clones to show the selected clones. The original image of this Western blot is shown in Supplementary Figure 1A. (**B**) Tumor growth of SW620 xenografts in athymic nude mice. A total of 10^6^ cells of each cell line (CDC42-wt1, parental SW620 cell line, CDC42-i1 and CDC42-i3) were injected subcutaneously in each flank of nude mice. Tumor volumes were determined twice a week for 35 days (*significantly different from control on day 35, *p*-value = 0.010, and ***significantly different from control on day 35, *p*-value = 0.004). (**C**) Heatmap showing the 190 differentially expressed genes using a fold-change cut-off of 1.5 in both groups of cells overexpressing CDC42 (CDC42-wt1 and CDC42-wt2) or with silenced CDC42 expression (CDC42-i1 and CDC42-i2) when compared to the parental SW620 cell line and then opposite differential expression between CDC42ov and CDC42i. (**D**) Gene Set Enrichment Analysis (GSEA) of the 190 differentially expressed genes. The upper panel shows GSEA analysis against the Molecular Signatures Database v4.0 (MSigDB) GO gene sets (C5)/GO cellular component collection (*p*-value < 0.05) and the bottom panel shows the top ten gene sets against the curated gene sets (C2)/canonical pathways collection ordered by count (number of genes from the 190 list included in each data set). See Supplementary Table 3 for the full list.

This *in vitro* model consists on cell clones with the stable expression of the wild type form of CDC42 as well as the genetic interference of CDC42 expression by shRNA in the adenocarcinoma colorectal cell line SW620 (Figure 1A). CDC42 overexpressing cells (CDC42ov) only showed a modest up-regulation compared to the parental cell line SW620 (Figure 1A and Supplementary Figure 1A), which already had high CDC42 basal levels compared to other CRC cell lines as well as to a primary colon fibroblast cell line, CCD-18Co (Supplementary Figure 1B). The cell clones generated for the genetic interference (CDC42 shRNA) reached between 40% (CDC42-i2, cell clone) and 70–85% (CDC42-i1 and CDC42-i3, cell clones) reduced protein expression (Figure 1A).

Figure 1B shows that the genetic down-regulation of CDC42 resulted in a significant reduction of tumor growth of the positive xenografts (see Supplementary Table 1 for tumor incidence) compared to the CDC42 wt1 or SW620 control cell lines as previously shown in other CRC cell lines [10–12]. These results consolidate the use of this cellular system as a powerful tool for the study of the CDC42-driven oncogenic transcriptional signature in CRC.

Next we carried out comparative gene expression arrays analysis in these cellular models to identify differentially expressed genes between CDC42 overexpressing cells and knock down clones. In total, 190 putative CDC42 target genes were identified (including 89 up-regulated and 101 down-regulated, Figure 1C and Supplementary Table 2). Gene Set Enrichment Analysis (GSEA) identified enrichment of GO terms relating to known CDC42 target pathways (Figure 1D, Top table), including cytoskeleton regulation and cell migration. Additionally, a role in epigenetic regulation by CDC42 also appears to be significant (term: chromatin). GSEA using the C2-CP collection (curated gene sets-canonical pathways, Figure 1D, bottom panel and Supplementary Table 3) further supports the role of CDC42 in epigenetic regulation with enrichment of multiple polycomb related gene sets (H3K27ME3, EED, SUZ1 and PRC2 targets) as well as a role in stem-cell related functions.

Complementary analyses using Ingenuity Pathway Analyses (IPA) identified “Cancer” as the most significant disease related term involving CDC42-dependent transcriptional deregulation (Supplementary Figure 2 and Figure 3). In keeping with a role of CDC42 in cancer onset and progression cellular growth, proliferation and cancer related pathways were significantly altered by modulation of CDC42 levels. Additionally, a plethora of signaling pathways are affected by CDC42 (Supplementary Figures 2 and 3) with potential effects on a diverse range of physiological processes. The top five networks identified with known molecular interactions that included most of the selected genes were: 1) Cell-to-cell signaling and interaction, cardiovascular system development and function, embryonic development; 2) Digestive system development and function, organismal injury and abnormalities, renal and urological disease; 3) Cell death and survival, embryonic development, cancer; 4) Cell-to-cell signaling and interaction, developmental disorder, hereditary disorder; 5) Amino acid metabolism, small molecule biochemistry, cellular growth and proliferation. Thus, the role of CDC42 in colorectal tumorigenesis could be caused by its function in transcriptional regulation in key genes involved in oncogenic processes.

**Figure 2:**
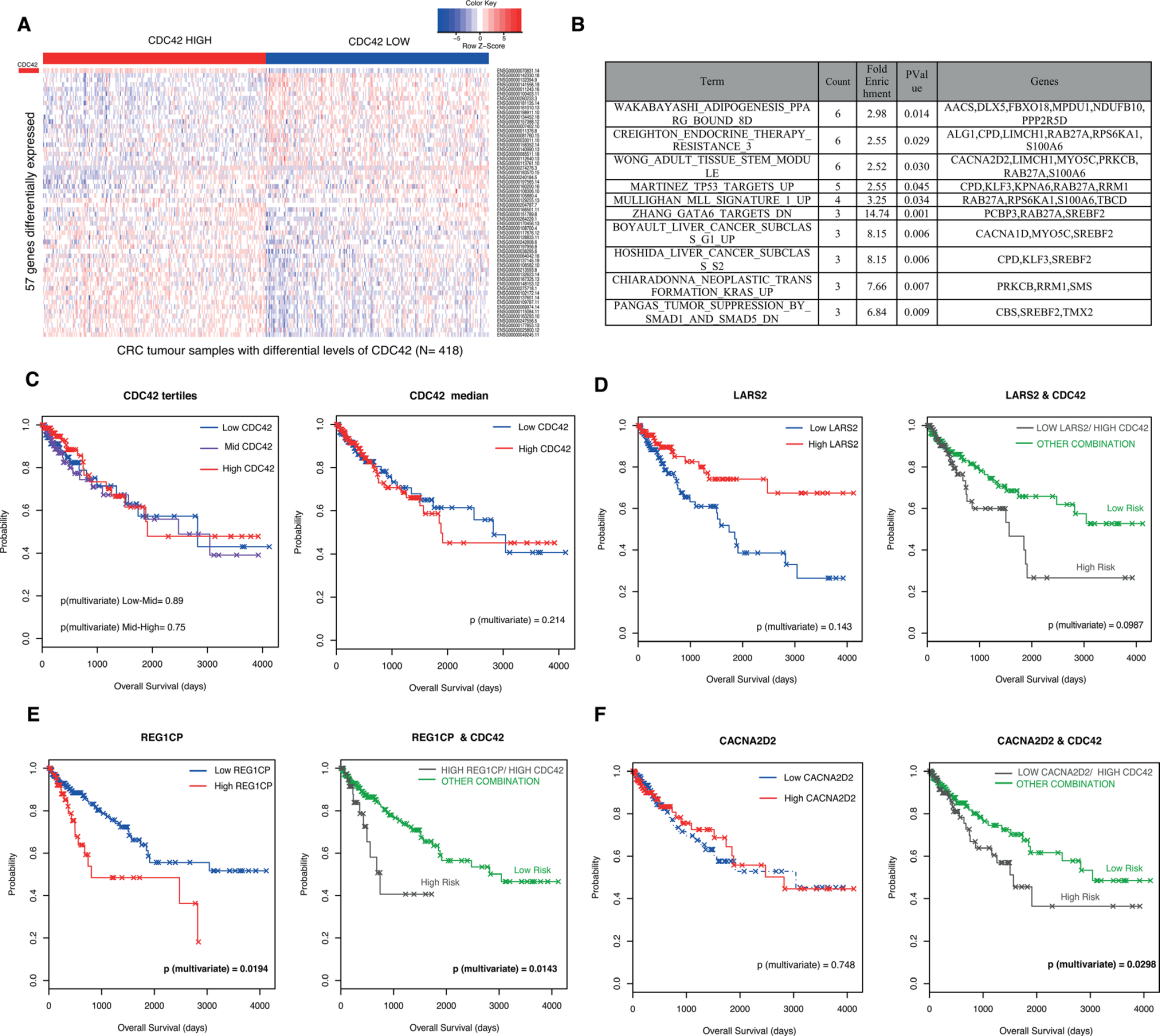
Validation of CDC42-transcriptional signature in CRC patients from The Cancer Genome Atlas (TCGA) Rectum Adenocarcinoma (READ) and Colon Adenocarcinoma datasets (COAD) (**A**) Heatmap of expression represented as Z-score of the 57 genes found to be correlated with CDC42 in the patient samples. Patients (*N* = 628) were stratified according to CDC42 expression levels and the top and bottom tertiles are shown in this heatmap. CDC42 expression levels are indicated in the first row (marked as a red rectangle). The top tertile is called “CDC42 HIGH” (red bar) and the bottom tertile is represented as “CDC42 LOW” (blue bar), this accounts for a total number of 418 patients. (**B**) Top ten gene sets from GSEA of the 57 differentially expressed genes analysis against the Molecular Signatures Database v4.0 GO gene sets against the curated gene sets (C2) /canonical pathways collection (*p*-value < 0.05) ordered by count. See Supplementary Table 5 for the full list. (**C**) Kaplan Meier (KM) plots for the overall survival analysis of 453 patients according to CDC42 levels. Left hand side (LHS) graph shows the survival curve stratifying the patients according to CDC42 tertiles (low, blue; mid, purple and high, red) and the right-hand side (RHS) plot according to CDC42 median (low, blue and red, high). Univariate and multivariate analysis were performed to evaluate the prognostic significance, and *p*-values from the multivariate analysis are shown in the graphs. A prognostic risk identifier was found in *LARS2* (**D**), *REG1CP* (**E**) *and CACNA2D2* (**F**) when combined with *CDC42* levels. The LHS KM plots show the survival analyses for these genes alone where high levels are represented in red and low levels are blue. The high-risk groups (gray) were then identified by taking the intersect of the *CDC42* high expression and *LARS2* low expression (D) *REG1CP* high expression (E) and *CACNA2D2* low expression (F) groups and compared to any other combination (green). *p*-values for multivariate analysis for each case are shown.

**Figure 3:**
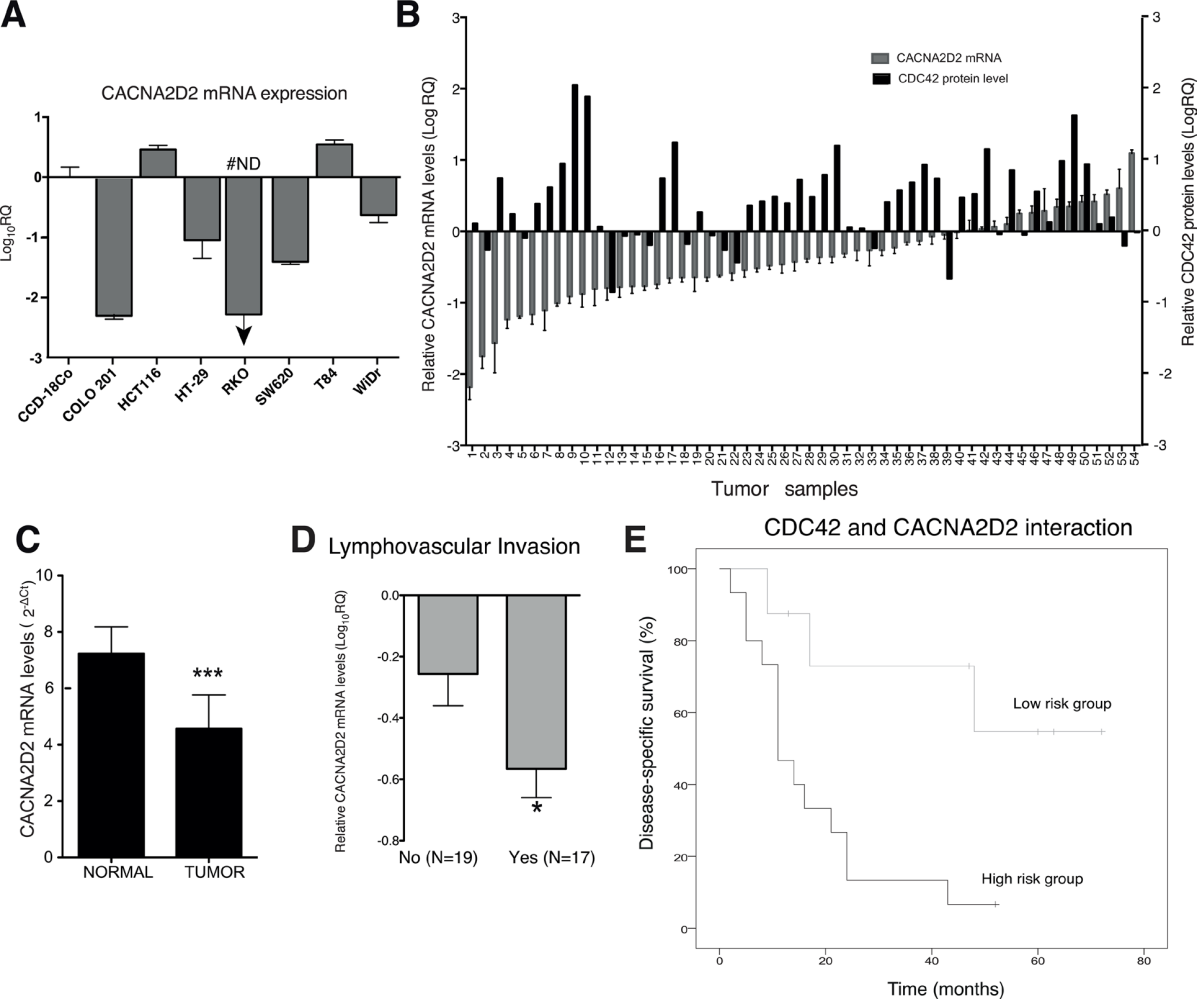
*CACNA2D2* expression in CRC and prognostic value in a CRC validation cohort (**A**) *CACNA2D2* gene expression was analyzed by qPCR in seven CRC cell lines and compared to the primary colon fibroblast cell line CCD-18Co, the data was normalized with 18S and relative to CCD-18Co (Log_10_RQ). #ND: not detected, CACNA2D2 mRNA was not detected in this sample by qPCR. (**B**) *CACNA2D2* mRNA expression (y-axis at the LHS) and CDC42 protein levels (y-axis at the RHS) in 54 CRC tumor samples are represented. CACNA2D2 and CDC42 expression levels are presented as the quantity of *CACNA2D2* gene in each tumor sample, normalized to an endogenous reference, 18S for *CACNA2D2* and the housekeeping protein GAPDH in the case of CDC42 and relative to its corresponding matched normal tissue for each patient. (**C**) Mean expression of *CACNA2D2* gene in normal and tumor tissues (***p-value < 0.0001). Data are presented as mean × 10^7^ ± SEM. (**D**) Mean expression of *CACNA2D2* gene in tumor tissues according to presence of lymphovascular invasion. *, significantly different from no presence of invasion (*p*-value = 0.03). (**E**) Disease-specific survival of the patients according the new proposed classifier based on *CACNA2D2* and CDC42 levels. Low risk group includes patients with Dukes´ C and D with low levels of CDC42 and moderate to high levels of *CACNA2D2* and the high risk group corresponds to Dukes´ C and D patients with high levels of CDC42 and low levels of *CACNA2D2*.

### Validation of the CDC42-transcriptional signature in CRC patient samples

To further validate this CDC42-driven transcriptional signature in the context of colorectal cancer, we used RNAseq data for a total of 628 CRC patients from The Cancer Genome Atlas (TCGA) Rectum Adenocarcinoma (READ) and Colon Adenocarcinoma (COAD) datasets [18]. Patients were stratified according to their *CDC42* mRNA levels. The top and bottom tertiles (*N* = 418) were used to interrogate which of the 190 genes found in SW620 cells were differentially expressed concordantly with CDC42 (Figure 1C). A total of 57 genes showed differential expression between *CDC42* groups (adjusted *p-value* < 0.05, Figure 2A and Supplementary Table 4) in same direction as observed in the cell line array data. GSEA of these genes clearly revealed their involvement in cancer-related pathways (Figure 2B and Supplementary Table 5), including p53 targets, liver cancer proliferation signatures, and KRAS neoplastic transformation. Altogether, these findings identify the most significant genes that correlate with CDC42 in CRC globally and independently of the particularities of a single colorectal cell line.

### Prognostic value of CDC42-transcriptional signature

We next explored if this transcriptional signature offered any advantage over the prognostic value for CDC42. First we analyzed the prognostic capacity of *CDC42* mRNA levels. Figure 2C shows that *CDC42* expression was not significantly associated with overall survival when the patients were segregated according to *CDC42* levels (tertiles or median). Next, we took the 57 genes concordantly differentially expressed with CDC42 (Supplementary Table 4) in the 453 TCGA CRC patients with sufficient clinical data and analyzed their prognosis value alone or in combination with *CDC42*. From this analysis, we found that the expression of three genes, *CACNA2D2, LARS2* and *REG1CP* render improved prognostic value when combined with *CDC42* (Figures 2D–2F), where in the case of *LARS2* and *REG1CP* there was only a modest improvement. Figure 2D shows that patients with low levels of *LARS1* and high levels of *CDC42* (*n* = 137) have worse prognosis than patients with any other combinations (*p*-value = 0.0987, right graph), this group was defined as “high risk group”, although this was only a trend and did not reach significance. The high risk group was able to predict prognosis better than using *CDC42* alone (Figure 2C, *p*-value = 0.214) as well as *LARS2* alone (Figure 2D left graph, *p*-value = 0.143). A similar scenario was found in the case of *REG1CP*, where a high risk group was defined as high *REG1CP* and high *CDC42* and significantly predicted a worse prognosis for this group of patients (Figure 2E, left plot, *p*-value = 0.0143). This prognostic identifier showed a slight improvement compared with *REG1CP* levels alone (Figure 2E right graph, *p*-value = 0.0194), and in both cases this prediction was significant. The best improvement of the prognostic value was observed in *CACNA2D2* gene; patients with a combination of high *CDC42* and low *CACNA2D2* had a significantly poorer prognosis (Figure 2F, right graph, *p*-value = 0.0298), while the levels of *CACNA2D2* alone were not associated with prognosis (Figure 2D, left graph *p*-value = 0.748).

Additionally, we found that the expression levels between *CDC42* and *CACNA2D2, LARS2* and *REG1CP* were significantly correlated in the tumor samples (Spearman's correlation *r* = −0.2507655 and *p*-value = 1.84e–10 for *CACNA2D2*; *r* = −0.2097521 and *p*-value 1.207e–07 for *LARS2* and *r* = 0.08323233 and *p*-value = 0.03705 for *REG1CP*) in agreement with our array data in SW620 cells.

### *CACNA2D2* is down-regulated in CRC and it is a better prognostic predictor when combined with CDC42

*CACNA2D2* is a tumor suppressor gene in several types of cancer [19, 20], however its role in CRC or correlation with CDC42 has not been described yet. *CACNA2D2* mRNA levels were analyzed in an array of CRC cell lines and compared to a primary colon fibroblast cell line, CCD-18Co (Figure 3A). We found that *CACNA2D2* was down-regulated in five out of 7 CRC cell lines (71%), supporting a potential role as TSG in this tumor type.

In order to confirm the possible clinical relevance of *CACNA2D2* and also its correlation with CDC42 in CRC, we validated our results in a independent third CRC cohort of 54 patients where we had previously analyzed CDC42 protein levels [8] (Table 1 and Figure 3B). *CACNA2D2* mRNA expression was found to be significantly decreased in colorectal carcinomas when compared to their corresponding matched normal colon tissues for 33 of 54 samples (61%) (Figure 3B and 3C, p*-value* < 0.0001). In addition, we found that higher down-regulation of *CACNA2D2* was significantly correlated with the presence of lymphovascular invasion (*p*-value = 0.03, Figure 3D). Next, the role of *CACNA2D2* as a prognostic factor in CRC patients was analyzed similarly as we did with the TCGA cohorts. Patients categorized by *CACNA2D2* levels using the median did not show any relevance with prognosis (data not shown) as we previously observed in the other cohorts (Figure 2D, left graph). We then looked if *CACNA2D2* prognostic significance improved when combined with CDC42. We found that only the patients of the poorest prognosis groups, Dukes’ C and D stages, showed a significant improvement in the prognostic value when these two genes were combined (Figure 3E and Table 2). Patients with high CDC42 (> 1.5 fold) and low *CACNA2D2* (lowest quartile, p25) showed poorer prognosis than the rest of the patients within the Dukes’ C and D stages, this group was defined as “high risk group” (HR4.68; 95% CI, 1.29–16.92; *p-value* = 0.019; Table 2).

**Table 1:** Clinical and pathological characteristics of patients from the CDC42 CRC cohort

Median age: 70.5 years, range 48–86	
	Number of patients (%)
**Gender**	
Female	18 (33.3)
Male	36 (66.7)
**T**	
T2	5 (9.3)
T3	40 (74.1)
T4	9 (16.7)
**Grade**	
Well/Moderately differentiated	40 (74.1)
Poorly differentiated	14 (25.9)
**Location**	
Colon	41 (75.9)
Rectum	13 (24.1)
**Dukes Stage**	
A	4 (7.4)
B	26 (48.1)
C	18 (33.3)
D	6 (11.1)
**Lymphovascular invasion**	
No	19 (35.2)
Yes	17 (31.5)
NA	18 (33.3)

NA not available.

**Table 2:** Uni-and multivariate analyses for disease-specific survival of Dukes´C and D patients

	Univariate analysis			Multivariate analysis		
	HR	95% CI	*p*-value	HR	95% CI	*p*-value
**T stage pT4 vs. pT3**	3.93	1.37–11.28	**0.011**	0.637	0.37–1.10	0.106
**Classifier**	4.68	1.29–16.92	**0.019**	3.662	0.94–14.28	**0.062**

The statistical correlation between CDC42 and *CACNA2D2* in the tumor samples was also investigated. Pearson's correlation test and the non-parametric Spearman correlation rank or Kendall tau correlation tests showed no direct correlation between CDC42 and *CACNA2D2* in the clinical samples (data not shown). This could be due to the small number of patients used in this cohort (*n* = 54 patients) compared to our TCGA cohort (*N* = 628), in which we did see a significant correlation. However, Cox regression analyses showed a significant interaction between *CACNA2D2* and CDC42 in the outcome of patients with regional lymph node or distant metastasis with CDC42 overexpression and reduced expression of *CACNA2D2* (*p*-value = 0.005), suggesting that these two molecular markers interact affecting the outcome of the patients.

In conclusion, the combined analysis of CDC42 and *CACNA2D2* expression levels provides a compelling tool for a better identification of patients at higher risk of mortality, especially for CRC patients in later stage disease.

### CDC42 regulates the expression of the putative tumor suppressor gene *CACNA2D2* in SW620 cells

In order to further validate *CACNA2D2* as a transcriptional target downstream CDC42 in CRC, we next studied whether its transcriptional silencing was CDC42-dependent in our *in vitro* SW620 cellular model (Figure 1).

**Figure 4:**
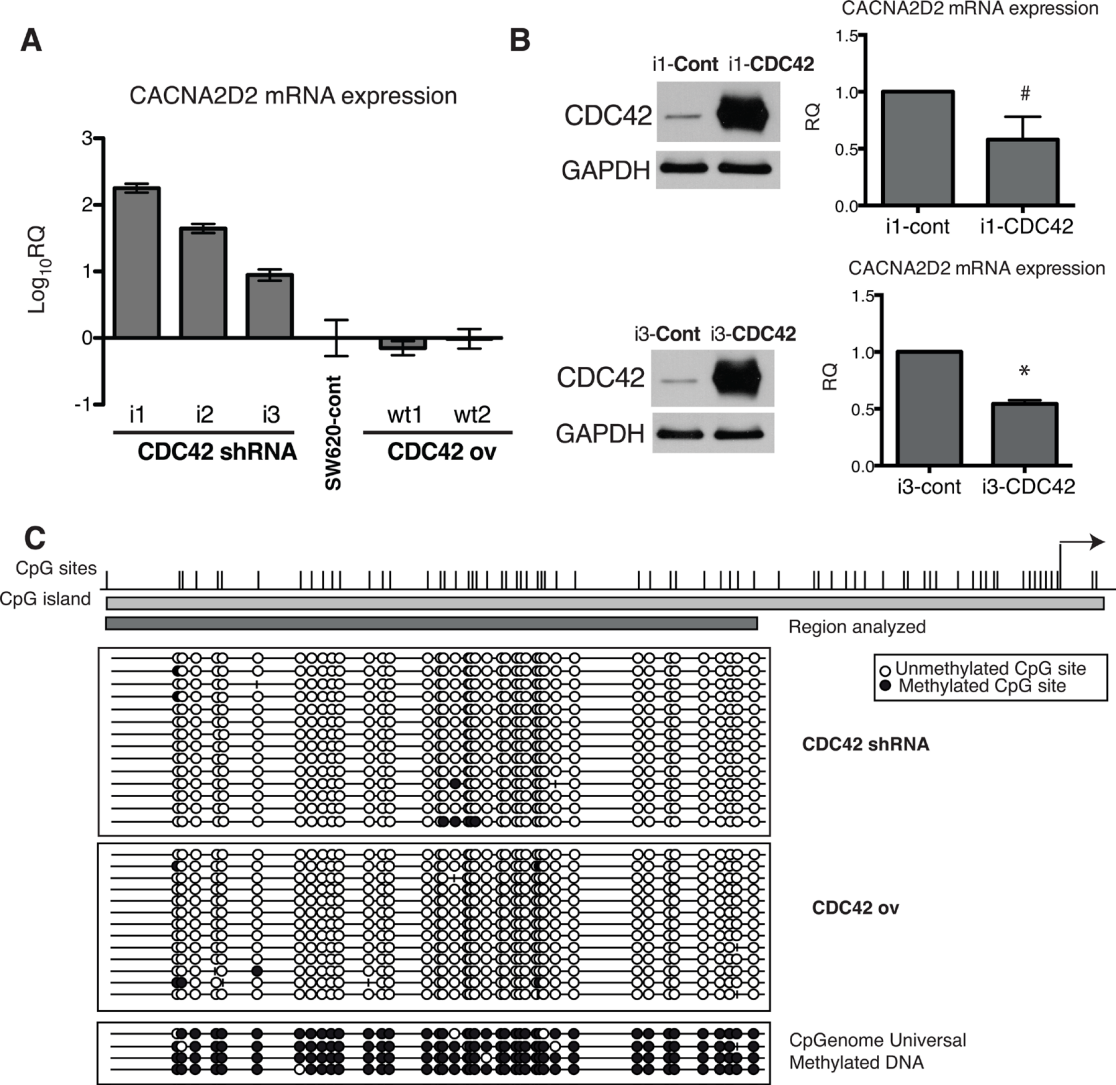
Transcriptional regulation of *CACNA2D2* by CDC42 in SW620 cells (**A**) *CACNA2D2* gene expression in our cellular model was determined by qPCR in the different stable cell lines and compared to the levels in the parental SW620 cell line by the 2^−ΔΔCT^ method. The results are shown as Log_10_ of relative quantity (RQ) of *CACNA2D2* in each cell line using control SW620 cells as reference. (**B**) LHS: CDC42 protein expression was determined by Western blot after transient overexpression of CDC42 (the original image of this Western blot is shown in Supplementary Figure 4A). Stable CDC42 interfering cell lines, i1 and i3, were transiently transfected with empty plasmid (Cont) or wild type form of CDC42 (CDC42) and CDC42 expression was determined at 48 h post-transfection. RHS: *CACNA2D2* gene expression determined by qPCR after transient overexpression of CDC42 in i1 and i3 cell lines. Expression was normalized using 18S as reference. Data are presented as the quantity of *CACNA2D2* expression in the CDC42-i1 and CDC42-i3 cell lines transiently overexpressing CDC42 (i1-CDC42 and i3-CDC42, respectively) relative to the expression in the lines transfected with the empty plasmid as controls (i1-cont and i3-cont, respectively). ^#^*p*-value = 0.1, *p-value = 0.0001, *N* = 3. (**C**) Bisulfite sequencing analysis of *CACNA2D2* promoter region. Bisulfite maps determined by direct sequencing of individual clones show the density of methylated CpG sites (black circles) and unmethylated CpG sites (white circles) at individual CpG residues. Virtually all sites were fully methylated when sequencing. CpGenome Universal Methylated DNA was used as positive control. Representative results of SW620 cells with silenced CDC42 expression (CDC42 shRNA) and cells overexpressing CDC42 (CDC42 ov) are shown.

First, we validated *CACNA2D2* expression array data through qPCR (Figure 4A). Increased expression of *CACNA2D2* gene was observed after CDC42 knock down, while *CACNA2D2* expression in both SW620 control cell line and CDC42ov cells was similar (Figure 4A), most likely due to the modest increase in *CDC42* expression when compared to the parental cell line. To further strengthen these findings, we attempted to rescue the *CACNA2D2*-low phenotype by transiently overexpressing CDC42 in the CDC42 shRNA transfectants (i1 and i3) (Figure 4B and Supplementary Figure 4A). Overexpression of CDC42 was very high after 48h, and consequently a ∼50% of down-regulation of *CACNA2D2* was observed (Figure 4B).

We then explored the plausible molecular mechanism by which CDC42 controls *CACNA2D2* mRNA expression. We have previously reported that CDC42 is involved in the silencing of *ID4* through promoter hypermethylation in the SW620 cellular model [8] and *CACNA2D2* has been already suggested to be silenced through promoter DNA methylation [21, 22]. Therefore we tested if CDC42 is regulating this gene through an epigenetic mechanism. Analysis of the promoter region of *CACNA2D2* by bisulfite clonal sequencing showed no differences in DNA methylation levels between CDC42 overexpressing and CDC42 shRNA cells with all possible CpG sites unmethylated in all of the cases (Figure 4C) ruling out an epigenetic mechanism as the explanation by which CDC42 silenced *CACNA2D2*.

### CDC42 regulates the expression of *CACNA2D2* in tumor xenografts

**Figure 5:**
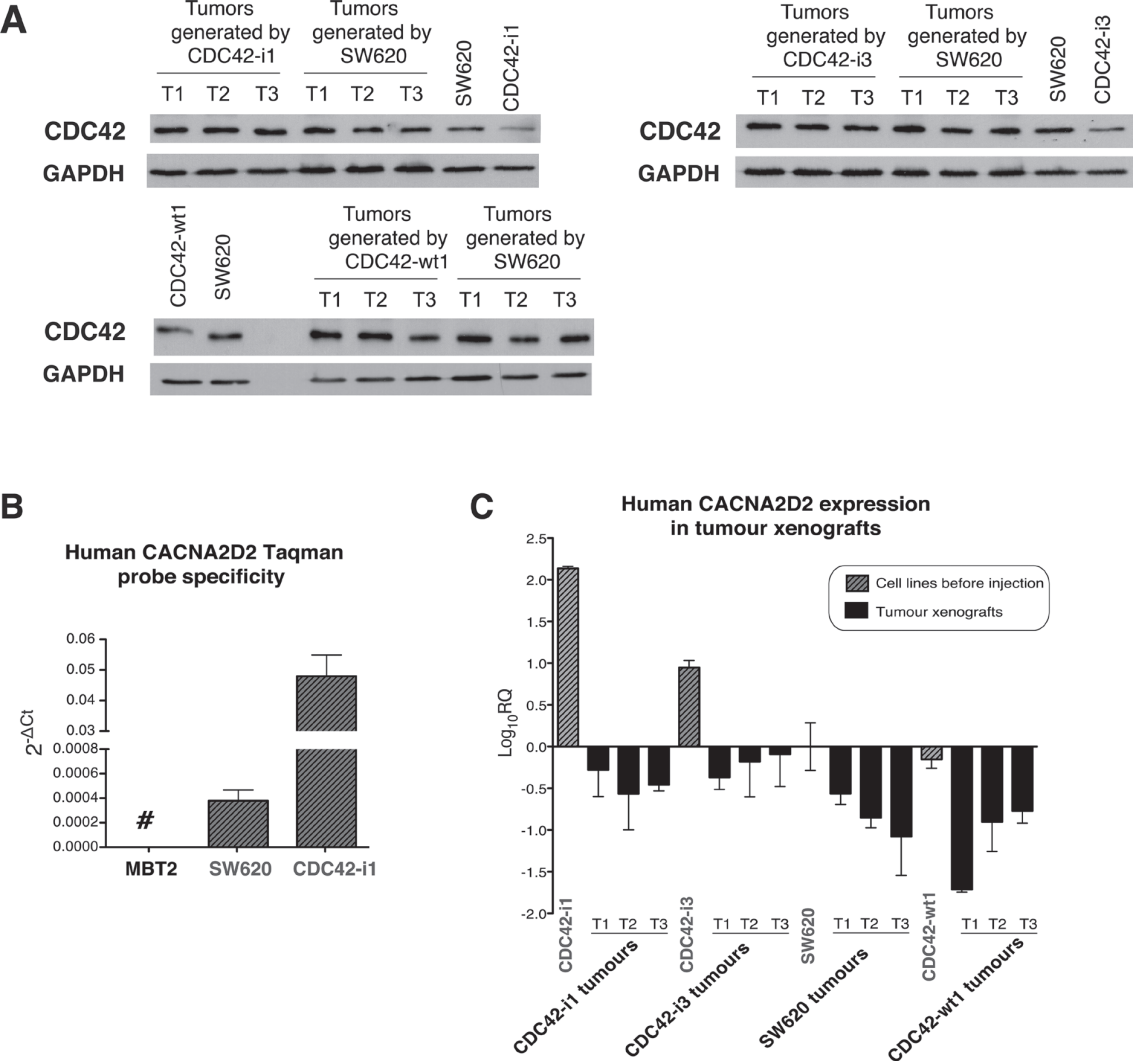
CDC42 and *CACNA2D2* expression in SW620 tumor xenografts (**A**) Analysis of total CDC42 protein levels in xenograft tumors generated by our SW620 cellular model of CDC42 knock down (CDC42-i1 and CDC42-i3), CDC42 overexpression (CDC42-wt1) and parental SW620 as well as in the cell lines prior injection. Western blot images of immunoblots stained with CDC42 and normalized using GAPDH as loading control. Three representative tumors (T1-T3) of each group are shown. The original images of these Western blots are shown in Supplementary Figure 4B. (**B**) Probe specificity for human *CACNA2D2* gene was tested using a murine cell line (MBT2) as negative control and two different human cell lines (SW620 and CDC42-i1 clone). (**C**) *CACNA2D2* gene expression was determined in the different tumor xenografts. *CACNA2D2* expression levels in cell lines before injection are shown as reference. *CACNA2D2* levels were normalized with the human-specific housekeeping gene *PGK1*. The results are shown as Log_10_ of relative quantity (RQ) of *CACNA2D2* in each sample using control SW620 cell line as reference

To further study the oncogenic link between the up-regulation of CDC42 and subsequent silencing of *CACNA2D2 in vivo*, we further characterized the tumor xenografts generated by CDC42 (Figure 1B) for CDC42 and *CACNA2D2* expression. First, we measured the protein expression levels of CDC42 in the tumor xenograft tissues (Figure 5A and Supplementary Figure 4B). Surprisingly, tumors derived from CDC42 shRNA cells exhibited CDC42 expression comparable to that of parental SW620 (representative examples shown in Figure 5A and Supplementary Figure 4B), suggesting that recovery of CDC42 expression is strictly required for tumor growth *in vivo*. Thus, we propose a sub-population of CDC42 shRNA cells was able to re-express endogenous CDC42 leading to tumor growth. Reactivation of CDC42 in the knock down cells is likely a major barrier to tumorigenesis and may account for the significant delay in tumor growth observed in these samples (Figure 1B). In the CDC42-wt1 group, CDC42 expression was comparable to that of the parental cells (Figure 5A and Supplementary Figure 4B) and subsequently, no delay in tumor formation was observed. Altogether, these results suggest that high levels of CDC42 are critical for tumor generation and progression *in vivo* in CRC.

Next, *CACNA2D2* mRNA levels were measured by qPCR in the generated tumors. As xenograft samples may contain an unknown mix of mouse and human cells, we assayed murine tissue contamination in our samples using a Taqman probe from the mouse-specific murine β-actin, and a housekeeping gene (Supplementary Figure 5A). Only residual levels of murine tissue were detected in xenograft samples when compared to a control xenograft derived from the mouse bladder cancer MBT2 cell line. These results demonstrated that our tumor samples were primarily represented by the injected human cell lines. Furthermore, a 100% human specificity was shown for the Taqman probe for human *CACNA2D2* gene as no expression was observed in the mouse MBT2 cell line (Figure 5B). To adjust *CACNA2D2* expression to the total amount of human content from the xenografts, we used the human-specific housekeeping gene *PGK1* (Supplementary Figure 5B), which also showed 100% specificity to human. Thus it is unlikely that murine contamination is likely to confound our findings.

*CACNA2D2* normalized levels were determined in xenograft tissues from SW620, CDC42-wt1, CDC42-i1 and CDC42-i3 tumors, along with the same cell lines prior injection (Figure 5C). Interestingly, when compared to CDC42 shRNA cells before injection, *CACNA2D2* expression was down-regulated in the CDC42 shRNA derived tumors to levels comparable to the parental CDC42-wt1 cell lines before injection and their corresponding tumor samples (Figure 5C). This result is in accordance to the loss of CDC42 interference observed after the *in vivo* experiment (Figure 5A) and to the reversion of the oncogenic phenotype induced by the presence of CDC42 (Figure 4B).

Altogether these findings demonstrated that CDC42 silences the TSG *CACNA2D2* in SW620 cells *in vitro* and *in vivo* confirming a transcriptional molecular link between the tumorigenic effect of CDC42 and the silencing of the TSG *CACNA2D2* in CRC observed in patients.

## DISCUSSION

CDC42 is a small GTPase involved in multiple cellular functions whose aberrant expression or/and activity has been shown to be oncogenic in different cancer types [23, 24]. We have previously reported that CDC42 is overexpressed in colorectal cancer and silenced the TSG *ID4* through an epigenetic mechanism [8]. Our results have been reinforced by recent studies showing that CDC42 activation promotes adhesion and invasion of CRC cells [10] and that incipient intestinal tumor cells activate CDC42 as a crucial step in malignant progression [12].

A better understanding of the biological characteristics of CRC could improve both the assessment of prognosis and the ability to predict response to treatments. Thus, the aim of the present study was to identify genes and pathways transcriptionally controlled by CDC42 with biological and clinical relevance in CRC. To that end, gene expression profiling of a colorectal cancer cell model with modulation of CDC42 levels has revealed the main networks in which this GTPase could be involved. Expression of 190 genes were significantly altered by CDC42. Both GSEA and IPA analysis demonstrated a significant impact on a broad range of transcriptional networks related to cell migration and proliferation, in agreement with previously published work [10, 11, 13–16]. A plausible explanation is that CDC42 may have a broad spectrum effect due to its ability to regulate critical transcription factors involved in signaling of some of these biological processes [25–28]. Different external signals can initiate CDC42 transduction cascades by triggering different kinases that ultimately activate transcription factors including STAT3 and 5a, NFκB, E2F, SRF, cMyc, AP-1, ATF2, ELK, Max and Chop that ultimately affect a broad spectrum of cell functions (reviewed in [17]). Most of these signaling pathways are affected in cancer.

Interestingly, our analysis also identifies chromatin regulation by the polycomb group and stem-cell related functions as novel targets of CDC42. CDC42 has been previously associated with the histone methyltransferase and PRC2 member EZH2 in T-cells in the context of actin polymerization in the cytosol [29], so it is possible that CDC42 transcriptional network reflects changes related to the polycomb group due to this association in SW620 cells. In addition, a role for CDC42 in self-renewal and differentiation of adult intestinal stem cells has been previously reported [30]. In this study, conditional ablation of *Cdc42* in the mouse intestinal epithelium resulted in the formation of large intracellular vacuolar structures containing microvilli in epithelial enterocytes, a phenotype reminiscent of human microvillus inclusion disease, characterized by severe nutrient deprivation. Concordantly, another study has shown that the inhibition of CDC42 blocks the formation of intestinal tumors and it is highly expressed in intestinal tumor stem cells [12].

Our newly discovered CDC42-transcriptional signature was next tested in 628 CRC patients, where we found that the expression of 57 genes correlated with CDC42 levels in the tumor samples in the same direction as seen in SW620 cells. GSEA showed that these genes were mostly related to cell proliferation pathways and KRAS neoplastic transformation. Approximately 30-50% of colorectal tumors are known to have a mutated *KRAS* gene. So our results suggest that CDC42 oncogenic effect might be enhanced in the context of *KRAS* mutated tumors. Indeed SW620 CRC cell line has *KRAS* mutated and KRAS has been previously shown to signal its oncogenic effect through AKT and CDC42 [31, 32]. In conclusion the genes regulated by CDC42 in CRC account for its role in proliferation and could be dependent on KRAS mutation status. However further investigation is required to establish this relationship in CRC.

Altogether, our study supports an oncogenic role of CDC42 in CRC by the transcriptional deregulation of key genes and pathways involved in several cancer-related processes.

While we have previously shown that high CDC42 is associated with less differentiated tumors [8], its prognostic value had not been tested in CRC. CDC42 levels in tumor samples did not correlate with prognosis, however we found that three genes have an improved prognostic value when combined with CDC42 status: *LARS2, REG1CP* and *CACNA2D2*. Inactivation of LARS2 (leucyl-tRNA synthetase in mitochondria) has been described in nasopharyngeal cancer cells [33], and dysregulation of aminoacyl-tRNA synthesis has also been widely linked to tumorigenesis [34], however this is the first report showing a transcriptional link between CDC42 and LARS2 in CRC. *REG1CP* (Regenerating Family Member 1 Gamma) is a pseudogene and is affiliated with the long non-coding RNA class, its physiological function is still unknown, so to our knowledge this is the first report to show its transcriptional association with CDC42 and its significant correlation with prognosis in CRC. *CACNA2D2* gene, a subunit of the voltage-dependent calcium channel complex, is located in the 3p21.3 chromosomal region, a known tumor suppressor gene cluster, that also includes *LARS2* [35]. A reduced expression of *CACNA2D2* has been described in lung cancer [36], gliomas [21], nasopharyngeal [22], head and neck squamous cell carcinoma [37] and cervical carcinoma [38]. Our study is the first report that demonstrates the direct effect of CDC42 on the transcriptional silencing of *CACNA2D2* gene. Prognostic implications of *CACNA2D2* have been reported in head and neck squamous cell carcinoma [37] and cervical carcinoma [38]. Here we also describe for the first time the clinical relevance of this gene in colorectal cancer. The transcriptional correlation and clinical significance of these three genes with CDC42 further supports our proposed novel role for CDC42 in the regulation of oncogenic transcriptional signatures in CRC.

Among these three genes, *CACNA2D2* showed the best correlation with CDC42 as well as the best improvement on the prognostic value when combined with CDC42, so we took this gene to perform further validation of its clinical relevance in a third CRC cohort as well as to further perform *in vitro* and *in vivo* studies its relationship with CDC42. Here we propose the combined use of CDC42 and *CACNA2D2* as a new prognostic classifier. In this validation cohort, survival analysis was intentionally restricted to the subgroup of patients with Dukes´ C and D tumor, where a significant interaction between the expression of CDC42 and *CACNA2D2* was found. This subgroup represents the most aggressive subgroup in which tumors show metastatic spread to lymph nodes or distant organs. A low risk group corresponding to patients whose tumors showed low levels of CDC42 and moderate to high levels of *CACNA2D2* was identified. Thus, this new classifier allows the identification of a group of patients with a higher risk of death.

Contrary to what was found in the case of the TSG *ID4* [8], CDC42-dependent *CACNA2D2* silencing was not due to DNA hypermethylation of the promoter, suggesting that the reduced expression may be associated with other mechanisms such as polycomb-mediated repression, mutation of the promoter region or dysfunction of transcription factors. It has been recently shown that the activity of pro-survival factors downstream of CDC42, such as PAK1 is increased in CDC42-driven colorectal tumorigenesis [12, 39] and these cascade pathways can regulate transcription through transcription factors like NFκB [40] and STAT3 [41]. CDC42 is able to activate several transcription factors, such as STAT3, NFκB and SRF [25–28], thus it might be possible that CDC42 controls the transcription of *CACNA2D2* gene through those or different transcription factors. However further research is needed to identify the specific molecular players in this CDC42-mediated signaling pathway.

Here we show for the first time a new oncogenic feature of CDC42, which is its ability to regulate the transcription of genes and pathways biologically and clinically relevant in CRC. Thus, CDC42 could be used as a therapeutic target for selective colorectal cancer intervention. In fact, recently some specific small molecules have been developed that specifically inhibits CDC42 [11, 42–44], providing a new means to study CDC42 and the cellular processes under its control. In keeping with the results presented here, one of these molecules, AZA197 suppressed primary colon cancer growth *in vivo* and prolonged survival in SW620 tumor xenografts, indicating the therapeutic potential of this inhibitor based on targeting CDC42 GTPase activity in colorectal cancer.

## MATERIALS AND METHODS

### Cell lines and cellular model

The following colorectal cancer cell lines were used in this study: SW620, WiDr, HT-29, HCT116, RKO, COLO 201 and T84 and the primary colon fibroblast cell line CCD-18Co. All cell lines were purchased from the American Type Culture Collection (ATCC). All these cell lines were passaged for less than 6 months after receipt. ATCC routinely performs STR analysis on its human cell lines. The cell lines were grown in the following media: DMEM (Gibco) in the case of CCD-18Co, HT-29, T84 and SW620 cells; EMEM (Gibco) for WiDr and RKO cells; RPMI (Gibco) for COLO 201 cells and McCoy´s 5A (ATCC) for HCT116 cells. All media were supplemented with 10% of Foetal Bovine Serum (FBS), 2 mM de glutamine, 0.5 μg/ml de fungizone y 100 U/ml de penicillin-streptomycin (all from Gibco).

The stable cellular model for the overexpression and knock down of CDC42 in SW620 cells has been previously reported [8]. Briefly, SW620 cells were transfected with Lipofectamine Plus Reagent (Invitrogen, Carlsbad, CA) according to the manufacturer´s instructions using pcDNA3B-wtCdc42 and pSUPER shRNA plasmids. SW620 cell line was authenticated for the last time in May 2016 by GeneMapper v3.7 (Applied Biosystems).

### Tumour xenograft establishment

Six-week old female athymic BALB/c nude mice were obtained from Charles River Laboratories. Animals were inoculated subcutaneously with 1 × 10^6^ cells in both flanks (eight animals per group, see also Supplementary Table 1). Tumor volumes were estimated twice a week and calculated as (length × width^2^)/2 using micrometer calipers. Thirty-five days later mice were euthanized, and tumor tissues were snap frozen in liquid nitrogen, and stored for future analysis. All animal procedures were approved by the Ethical Committee of Animal Experimentation (CEEA-CNB) of Centro Nacional de Biotecnologia (CNB-CSIC, Madrid, Spain) in accordance with national and international guidelines and with the Royal Decree (RD 1201/2005). Permit number: CEEA-CNB: 080047.

### Protein extraction from cell lines and tissues and Western blot

For protein analysis, cells were harvested, washed twice with ice-cold PBS, and incubated in ice-cold lysis buffer [50 mMTris-HCl (pH 7.5), 10 mM Na_4_P_2_O_7_, 50 mM NaF, 5 mM EDTA, 0.5% Triton X-100 (v/v), 0.5% sodium deoxycholate (w/v)] along with phosphatase and protease inhibitors.

In the case of xenograft samples, proteins were extracted from 30-50 mg frozen tissues. Samples were homogenized and lysed in ice-cold buffer containing 25 mM HEPES (pH 7.5), 1.5 mM MgCl_2_, 0.2 mM EDTA, 0.3 M NaCl, 20 mM β-glycerophosphate and 0.1% Triton X-100 (v/v) supplemented with phosphatase/protease inhibitors and benzonase. Nuclei and detergent-insoluble material were removed by centrifugation at 14,000 rpm for 20 minutes at 4°C. Protein concentration was determined with Bradford Assay (Bio-Rad, Hercules, CA).

CDC42 expression in cell lines and xenograft tissues was determined by Western blot as previously described [8] using specific monoclonal antibodies for CDC42 (BD Transduction Laboratories, Lexington, KY), Tubulin (Sigma Chemical Co) and GAPDH (Chemicon International Inc., Temecula, CA, USA).

### Gene expression arrays and bioinformatic analysis

Differential gene expression induced through genetic manipulation of CDC42 levels in SW620 cell line was determined using Human19K Oligo Array from Center for Applied Genomics (University of Medicine of New Jersey). Microarrays hybridization protocol, signal detection and data normalization description have been previously reported [45]. The experiments were made using biological replicates (two different sets of clones for each genetic modification) and also experimental replicates (performing the whole microarrays twice). Specifically, genes differentially expressed were identified by using a fold-change cut-off of 1.5 in both groups of cells overexpressing CDC42 (CDC42ov) or with silenced CDC42 expression (CDC42i) when compared to the parental SW620 cell line and then opposite differential expression between CDC42ov and CDC42i (Supplementary Table 2).

Gene Set Enrichment Analysis (GSEA) was performed against the Molecular Signatures Database v4.0 (MSigDB) curated gene sets (C2) and GO gene sets (C5) Collections [46]. Enrichment was assessed by hypergeometric testing as implemented in the R stats package.

Ingenuity Pathways analysis software (IPA, Ingenuity Systems, www.ingenuity.com) was used to integrate the most significant biological pathways regulated by CDC42. A list of 190 differentially expressed genes was created (*p*-value < 0.05, fold change > 1.5 & < −1.5). This dataset containing gene identifiers and corresponding fold change was uploaded to define the functional networks of differentially expressed genes. The analysis of the 190 genes showed 24 genes of unknown function and the remaining 166 genes were further analyzed.

### Acquisition and survival analysis of the cancer genome atlas (TCGA) expression data

Processed RNA-seq data and clinical data for The Cancer Genome Atlas (TCGA) Rectum Adenocarcinoma (READ) and Colon Adenocarcinoma (COAD) [18] were obtained through the NIH Genomic Data commons data portal. Processed RNA-seq expression data was available for 628 tumors of which 460 had available clinical information. For the platform comparison between microarray and RNAseq data, Human19K Oligo Array annotation to ensemble gene ID was successful for 171 genes out of the original 190 genes. Differential expression analysis was performed using processed HTSeq counts data in R using the package DESeq2 [47].

Survival analysis was performed on the set of patients that had available clinical information and were aged < 90 years, leaving 453 patients. Stratification of patients into high and low expression groups was performed using upper quartile normalized FPKM values (obtained from TCGA) such that high expression patients were defined as having gene expression above the cancer population median. The high-risk population was then identified by taking the intersect of the *CDC42* high expression and *CACNA2D2* low expression, *LARS2* low expression and *REG1CP* high expression groups, respectively. Analysis of survival was performed using a Cox proportional hazards model as implemented in the R package survival [48, 49]. Analysis was performed with membership in high/low expression groups as the explanatory variable (univariate) and age, T classification, evidence of venous and or lymphatic invasion, gender, tumor type (Colon or Rectum) and stage (multivariate). Tumor stage was collapsed to just 2 categories of Stage 1 & 2 and Stage 3.

### Patient tissues and survival data from the CDC42 CRC cohort

Fresh-frozen tumor specimens from 57 colorectal adenocarcinomas were obtained from treatment naïve patients (36 men and 18 women) who underwent surgery at La Paz University Hospital of Madrid, between 2003 and 2006. All specimens were snap-frozen in liquid nitrogen immediately after surgery for storage at −80°C. This study was approved by the Ethics Committee of La Paz University Hospital and all patients gave informed consent. This patient cohort was previously used to analyze the expression levels of the protein CDC42 [8]. The CDC42 protein information has been also used in this study.

The RNA from 54 patients of this cohort met the quality requirements to proceed for cDNA and qPCR analysis. Clinical and pathological variables were determined and are summarized in Table 1. All patients had information on age, sex, grade, tumor location and lymphovascular invasion.

For the study of the prognostic classifier, an analysis based on disease-specific survival restricted to stage II and III tumors was performed. A new classifier was then developed to integrate the interaction of CDC42 high expression and *CACNA2D2* low expression and allow a classification of patients in good or poor prognosis groups based on disease-specific survival restricted to Dukes´ C and D tumors. Survival curves of the patients in the different risk groups were compared using the Kaplan-Meier method and analyzed by the log-rank test. Cox proportional-hazards models were used to estimate survival distributions, hazard ratios and interaction analysis.

To evaluate if this new proposed classifier might constitute an independent prognostic factor, clinical and histopathological data were included in both univariate and multivariate Cox regression analyses. Only T stage was found significant in the univariate analysis and therefore used as covariate for the multivariate adjustment. The univariate associations between *CACNA2D2* gene expression and the different clinical-pathological parameters were assessed by means of the Mann-Whitney or Kruskal-Wallis tests.

The statistical analyses were performed using SPSS software, version 19 (Inc, Chicago, Illinois).

### Statistical analysis

Pearson's correlation test and the non-parametric Spearman correlation rank or Kendall tau correlation tests were used to study the relationship between *CDC42* and *LARS2*, *REG1CP* and *CACNA2D2* results.

All statistical analyses were two-sided and considered significant if *p*-value < 0.05.

### RNA extraction from cell lines and tissues and qPCR

Total RNA was prepared from the cell lines using the RNeasy Mini kit (Qiagen, Hilden, Germany) and prepared from xenograft and patient tissues using Trizol^®^ Reagent (Invitrogen) and cleaned up with RNeasy Mini kit (Qiagen) by following the manufacturer's instructions. Total RNA from each sample was quantified by the Nanodrop ND 1000 and RNA integrity was assessed by standard electrophoresis on agarose gel. First strand cDNA was synthesized from 1 μg of total RNA using the High-Capacity cDNA Archive Kit (Applied Biosystems) at 37°C for 2 h.

The expression of *CACNA2D2* and *CDC42* genes was quantified by real-time PCR. Each cDNA sample was analyzed in triplicate using the ABI PRISM 770 Sequence Detector (Applied Biosystems). 18S ribosomal RNA was amplified as reference gene for cell lines and clinical tissues. For normalization in the case of xenograft tissues, *PGK1* was used as reference gene and the contamination of murine tissue was determined after amplification of murine actin (*Actb)*. Probes used for amplification were purchased from Applied Biosystems as Taqman Gene Expression Assays (*CACNA2D2* Assay ID: Hs00195772_m1; *CDC42* Assay ID: Hs00741586_mH; 18S ribosomal RNA Assay ID: Hs99999901_s1; *PGK1* Assay ID: Hs99999906_m1; *Actb* Assay ID: Mm00607939_s1). Relative quantification of gene expression was calculated with the 2^−ΔΔCt^ method [50].

### Bisulfite clonal sequencing of *CACNA2D2* promoter region

Genomic DNA extraction, bisulfite modification and sequencing protocols have been described previously [8]. A semi nested PCR for *CACNA2D2* promoter region was performed. The first round of amplification was done in a 20 μl volume with 2 μl template and 1 μM F primer (5′-TTATTATTAAATTTGTGATTTTAGGTTTTAAGTT-3′), 1 μM R primer (5′-TCCCTACAACGCTAACTCCAAA-3′) and 2.5U AmpliTaq Gold polymerase (Applied Biosystems, Foster City, CA) for 30cycles at 95°C, 30 sec at 52°C, and 1 min at 72°C, followed by a 10-min final extension at 72°C. Of the first round of PCR, a 1 μl sample was used for the second, in a 50μl volume using the same conditions but containing 0.8μl of a nested F primer (5′-GTTTTTAAAAGGGTTATATATGTTTTTGTT-3′) and 0.8 μl of the R primer used in the first round of PCR. CpGenome^TM^ Universal Methylated DNA (Chemicon International) was used as positive control of fully methylated human genomic DNA.

### Transient transfection

For transient transfection, cells were seeded 48 h before transfection at a density of 3.5 × 10^6^ cells/well in 6-well plate and transfected with 2 μg of pcDNA3B-wtCdc42 plasmid using Lipofectamine Plus reagent (Invitrogen) according to manufacturer´s recommendations. The cells were harvested 48 h post-transfection, and overexpression was assessed by Western blot analysis.

## SUPPLEMENTARY MATERIALS FIGURES AND TABLES










