# Supplementary material for: Clinical relevance of the transcriptional signature regulated by CDC42 in colorectal cancer
Source: Oncotarget. 2017 Mar 1;8(16):26755–70. doi: 10.18632/oncotarget.15815 (PMC5432295; doi:10.18632/oncotarget.15815)
Supplement: Supplementary file 1 [file oncotarget-08-26755-s001.pdf]

Clinical relevance of the transcriptional signature regulated by CDC42 in colorectal cancer

Supplementary Materials

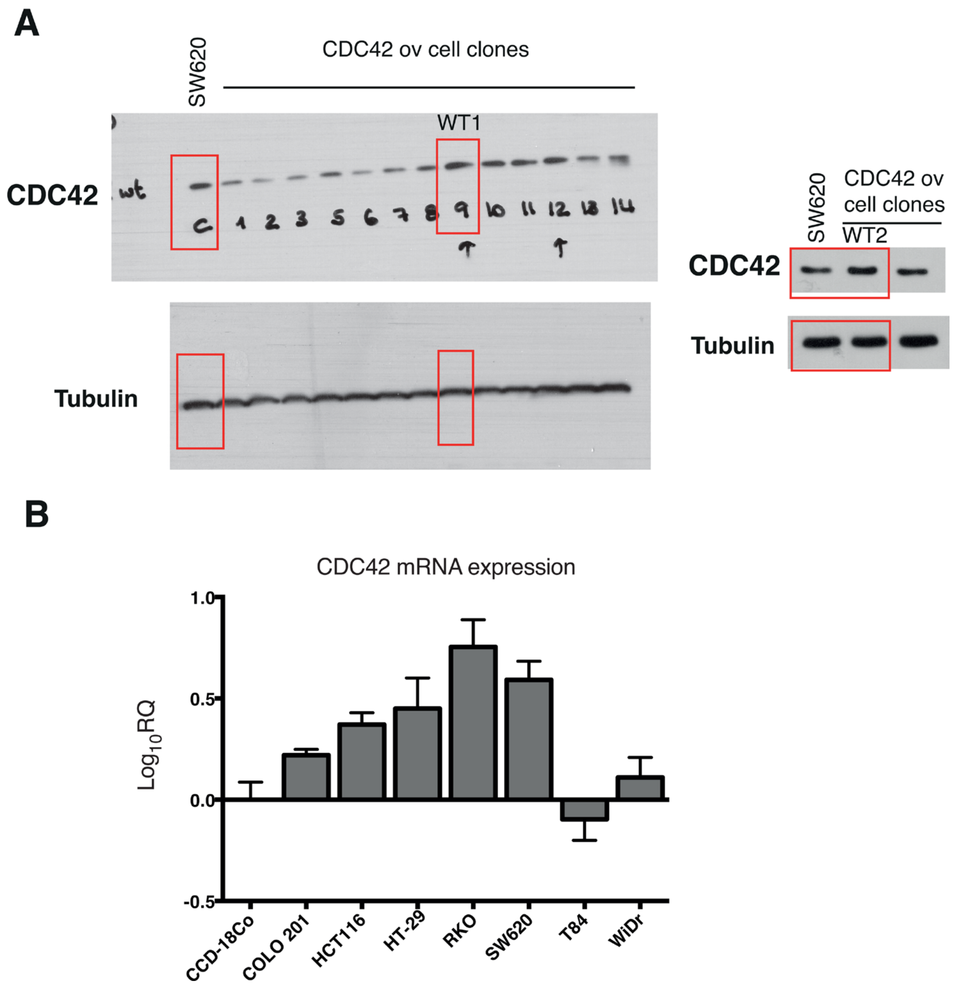

Supplementary Figure 1: (**A**) Original Western Blot images for CDC42 and Tubulin of the CDC42 overexpressing clones (CDC42 ov) used in this study. The red rectangle indicates the images taken to build Figure 1A. (**B**) *CDC42* mRNA expression was analyzed by qPCR in seven CRC cell lines and compared to the primary colon fibroblast cell line CCD-18Co, the data was normalized with 18S and relative to CCD-18Co (Log_{10}RQ).

Top Diseases and Bio Functions

Diseases and Disorders

Name	p-value range	# Molecules
Cancer	1.35E-02 - 1.74E-04	125
Organismal Injury and Abnormalities	1.35E-02 - 1.74E-04	130
Developmental Disorder	1.35E-02 - 1.75E-04	48
Neurological Disease	1.35E-02 - 1.75E-04	34
Cardiovascular Disease	1.35E-02 - 2.71E-04	22

Molecular and Cellular Functions

Name	p-value range	# Molecules
Cell Cycle	1.35E-02 - 4.56E-05	22
Cell Death and Survival	1.35E-02 - 4.56E-05	56
Lipid Metabolism	1.35E-02 - 8.24E-05	23
Molecular Transport	1.35E-02 - 8.24E-05	36
Small Molecule Biochemistry	1.35E-02 - 8.24E-05	35

Physiological System Development and Function

Name	p-value range	# Molecules
Endocrine System Development and Function	1.35E-02 - 2.01E-05	7
Organ Morphology	1.35E-02 - 2.01E-05	22
Embryonic Development	1.35E-02 - 4.56E-05	30
Tissue Development	1.35E-02 - 2.20E-04	34
Digestive System Development and Function	1.35E-02 - 5.29E-04	19

Top Networks

ID	Associated Network Functions	Score
1	Cell-To-Cell Signaling and Interaction, Cardiovascular System Development and Function, Embryonic Development	48
2	Digestive System Development and Function, Organismal Injury and Abnormalities, Renal and Urological Disease	38
3	Cell Death and Survival, Embryonic Development, Cancer	35
4	Cell-To-Cell Signaling and Interaction, Developmental Disorder, Hereditary Disorder	26
5	Amino Acid Metabolism, Small Molecule Biochemistry, Cellular Growth and Proliferation	26

Supplementary Figure 2: Ingenuity pathways analysis (IPA) summary of the top diseases and bio functions and networks transcriptionally regulated by CDC42. A total number of 190 genes were identified from the array analyses, 89 were up-regulated and 101 down-regulated by CDC42. Cancer and cancer-related functions are significantly overrepresented in the IPA analysis.

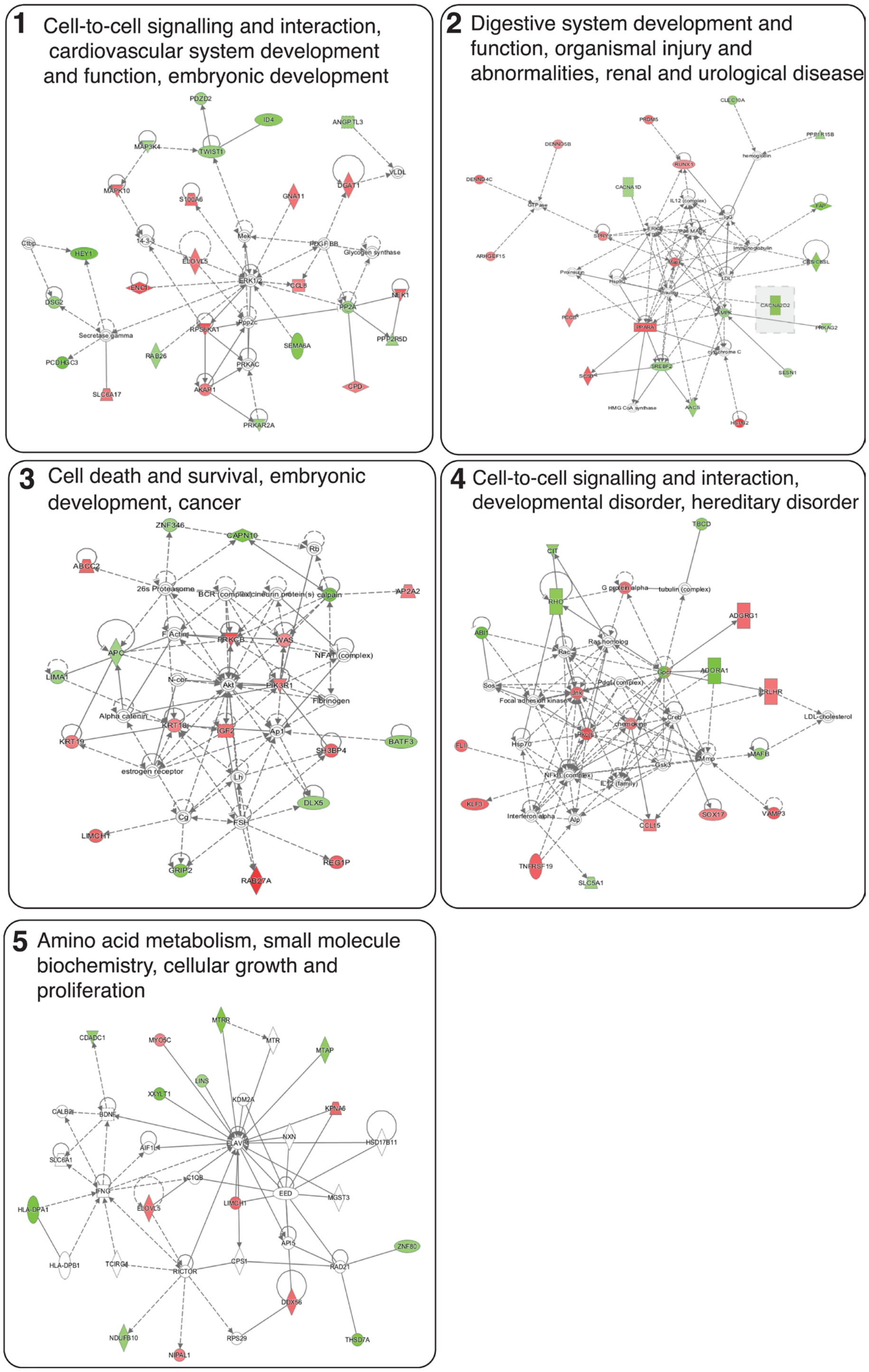

Supplementary Figure 3: Gene networks modulated by CDC42 in colorectal cancer. IPA was used to identify relevant gene networks. The top 5 relevant gene networks, including most of the differentially expressed genes, are shown. Red nodes represent up-regulated genes and green nodes represent down-regulated genes identified by microarray analysis. White nodes represent genes not regulated by CDC42 but related to these signalling pathways. Solid lines imply direct relationships between proteins; dotted lines imply indirect interactions. Relationships are primarily due to co-expression, but can also include phosphorylation/dephosphorylation, proteolysis, activation/deactivation, transcription, binding, inhibition, biochemical modification.

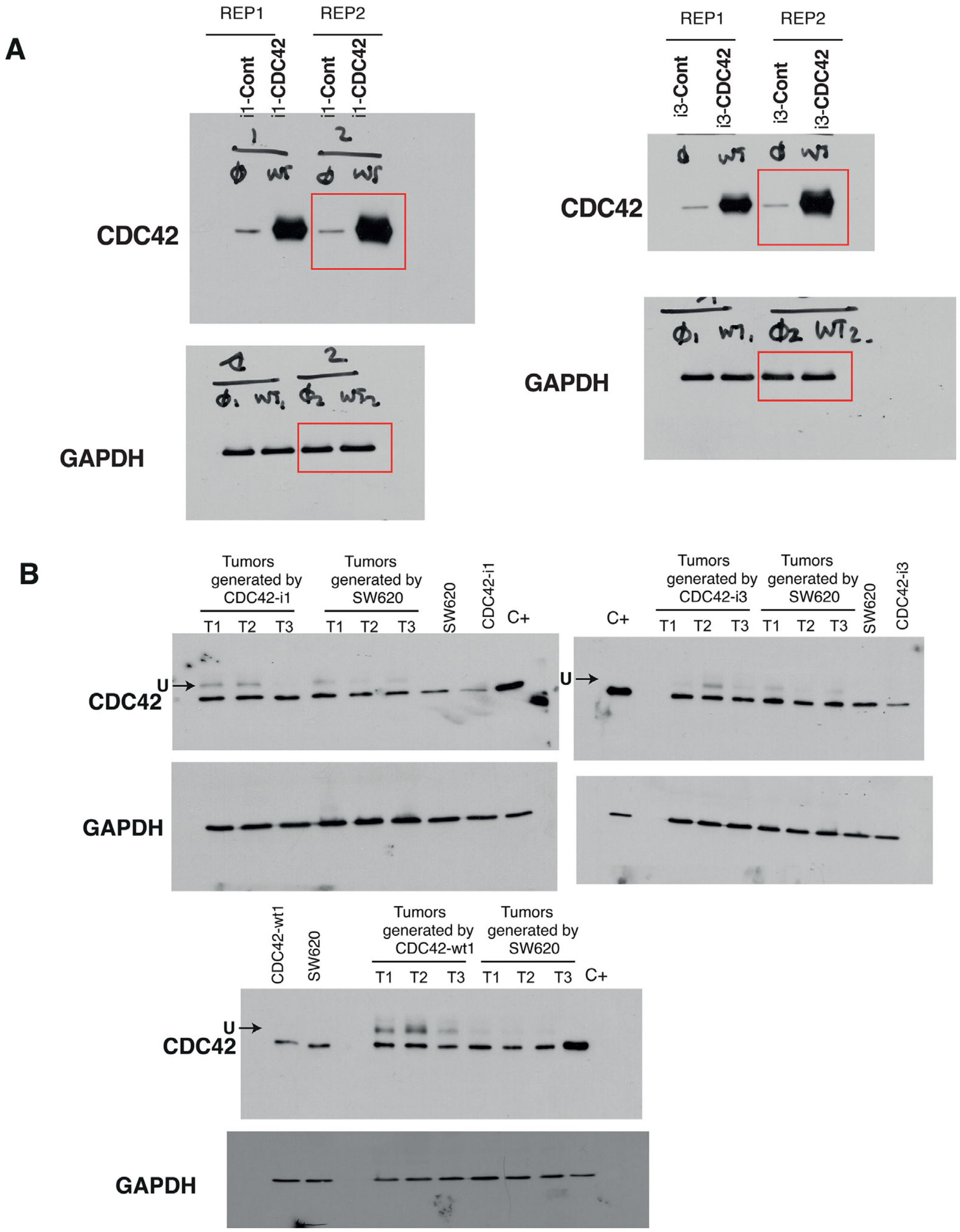

Supplementary Figure 4: (**A**) Original Western Blot images for CDC42 and GAPDH of the transient transfection of CDC42 in the SW620-CDC42-i1 and SW620-CDC42-i3 cells. The red rectangle indicates the images taken to build Figure 4B. (**B**) Original Western Blot images for CDC42 and GAPDH of the xenograft tumors generated from the different CDC42-clones. A positive control of HEK293T cells overexpressing CDC42 was used (C+). An unspecific upper band (U) appeared in some of the tumors analysed.

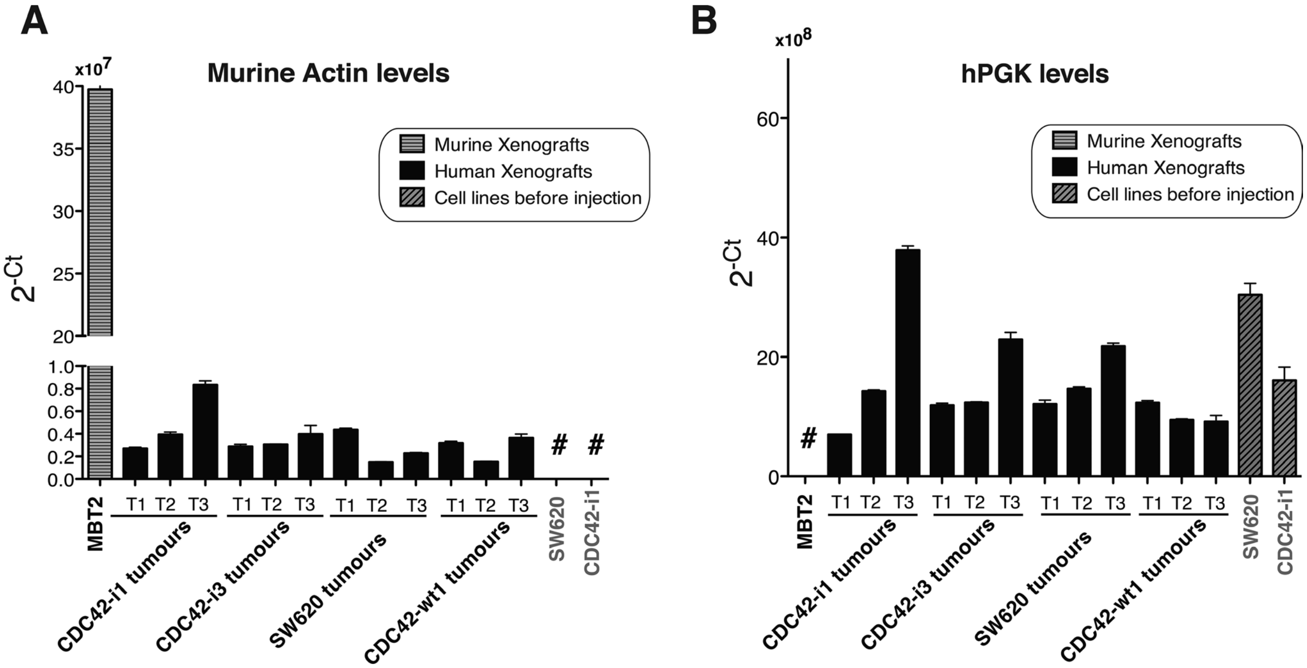

Supplementary Figure 5: Estimation of mouse contamination in the xenograft samples. For xenograft specimens, the percentage of mouse and human component was determined by qPCR measurement using species-specific probes, β-actin for mouse component (left panel) and *PGK1* for human component (right panel). MBT2 mouse cell line xenograft was used as positive control for mouse specificity and SW620 and CDC42-i1 cell lines were used as positive control of human specificity.

Supplementary Table 1: Tumor incidence of the CDC42-SW620 xenografts

GROUP	FLANKS INOCULATED	POSITIVE FLANKS`	TUMOR INCIDENCE (%)
**CDC42-i1**	16	4	25
**CDC42-i3**	16	16	100
**SW620-cont**	16	15	94
**CDC42-wt1**	16	13	81

Supplementary Table 2: List of differentially expressed genes (*p*-value ≤ 0.05) by CDC42 in SW620 cells. See Supplementary_Table_2

Supplementary Table 3: Full table of gene set enrichment analyses for curated gene sets/canonical pathways (*p*-value ≤ 0.05) for CDC42 regulated genes in SW620 cells. See Supplementary_Table_3

Supplementary Table 4: 57 genes correlated with CDC42 transcriptional signature in TCGA CRC patients (red: up-regulated genes, blue: down-regulated genes). See Supplementary_Table_4

Supplementary Table 5: Full table of gene set enrichment analyses for curated gene sets/ canonical pathways (*p*-value ≤ 0.05) for CDC42 regulated genes in TCGA datasets. See Supplementary_ Table_5
