# Supplementary material for: Clinical relevance of the transcriptional signature regulated by CDC42 in colorectal cancer
Source: Oncotarget. 2017 Mar 1;8(16):26755–70. doi: 10.18632/oncotarget.15815 (PMC5432295; doi:10.18632/oncotarget.15815)
Supplement: Supplementary file 2 [file oncotarget-08-26755-s002.docx]

**Table S2: List of differentially expressed genes (p-value < 0.05) by CDC42 in SW620 cells**

| Cdc42  int | Cdc42wt | Fold difference | Log2  Fold change | p-value | GeneBank accession  number | Ensembl Gene ID | HGNC  symbol | Description |
| --- | --- | --- | --- | --- | --- | --- | --- | --- |
| 1.30 | 0.50 | 0.38 | -1.40 | 0.001 | M27487 | ENSG00000231389 | HLA-DPA1 | major histocompatibility complex. class II. DP alpha 1 |
| 1.93 | 0.80 | 0.41 | -1.28 | 0.001 | AL161982 | ENSG00000185737 | NRG3 | neuregulin 3 |
| 0.53 | 1.46 | 2.77 | 1.47 | 0.001 | AF086295 | #N/A | #N/A |  |
| 0.38 | 1.27 | 3.33 | 1.73 | 0.001 | AK021986 | #N/A | #N/A |  |
| 0.69 | 1.62 | 2.36 | 1.24 | 0.001 | D50918 | ENSG00000125354 | SEPT6 | septin 6 |
| 1.79 | 0.56 | 0.32 | -1.67 | 0.001 | AF090888 | ENSG00000136754 | ABI1 | abl interactor 1 |
| 0.77 | 1.74 | 2.25 | 1.17 | 0.001 | NM_005777 | ENSG00000004534 | RBM6 | RNA binding motif protein 6 |
| 0.63 | 1.56 | 2.47 | 1.30 | 0.001 | NM_001541 | ENSG00000170276 | HSPB2 | heat shock 27kDa protein 2 |
| 1.36 | 0.57 | 0.42 | -1.25 | 0.002 | AK026890 | #N/A | #N/A |  |
| 1.43 | 0.65 | 0.45 | -1.14 | 0.002 | AF279656 | ENSG00000092421 | SEMA6A | semaphorin 6A-1 |
| 1.50 | 0.56 | 0.37 | -1.42 | 0.002 | NM_000674 | ENSG00000163485 | ADORA1 | adenosine A1 receptor |
| 0.47 | 1.05 | 2.24 | 1.17 | 0.002 | AY007106 | ENSG00000247556 | OIP5-AS1 | hypothetical protein LOC729082 |
| 0.60 | 1.39 | 2.32 | 1.21 | 0.002 | AK022893 | ENSG00000229191 | #N/A |  |
| 1.21 | 0.51 | 0.42 | -1.25 | 0.002 | AB023177 | ENSG00000005108 | THSD7A | thrombospondin. type I. domain containing 7A |
| 0.63 | 1.55 | 2.45 | 1.29 | 0.002 | L10151 | #N/A | #N/A |  |
| 0.61 | 1.28 | 2.11 | 1.08 | 0.002 | AF143327 | #N/A | #N/A |  |
| 0.54 | 1.15 | 2.13 | 1.09 | 0.002 | AK024912 | ENSG00000137601 | NEK1 | NIMA related kinase 1 |
| 0.51 | 1.17 | 2.28 | 1.19 | 0.002 | AF070584 | ENSG00000171953 | ATPAF2 | ATP synthase mitochondrial F1 complex assembly factor 2 |
| 1.74 | 0.61 | 0.35 | -1.50 | 0.003 | NM_012258 | ENSG00000164683 | HEY1 | hairy/enhancer-of-split related with YRPW motif 1 |
| 1.22 | 0.54 | 0.44 | -1.18 | 0.003 | AF254088 | #N/A | #N/A |  |
| 1.53 | 0.56 | 0.36 | -1.46 | 0.003 | AF131805 | ENSG00000206562 | METTL6 | methyltransferase like 6 |
| 1.76 | 0.74 | 0.42 | -1.25 | 0.003 | NM_004460 | ENSG00000078098 | FAP | fibroblast activation protein alpha |
| 0.50 | 1.12 | 2.24 | 1.16 | 0.003 | NM_002953 | ENSG00000117676 | RPS6KA1 | ribosomal protein S6 kinase. 90kDa. polypeptide 1 |
| 1.85 | 0.81 | 0.44 | -1.20 | 0.003 | AL110204 | ENSG00000181722 | ZBTB20 | zinc finger and BTB domain containing 20 |
| 0.54 | 1.13 | 2.11 | 1.08 | 0.003 | AK026072 | ENSG00000151789 | ZNF385D | zinc finger protein 385D |
| 1.44 | 0.50 | 0.34 | -1.54 | 0.003 | AK025042 | ENSG00000239205 | #N/A |  |
| 0.73 | 1.54 | 2.11 | 1.07 | 0.003 | AF113689 | ENSG00000167325 | RRM1 | ribonucleotide reductase catalytic subunit M1 |
| 0.84 | 1.66 | 1.98 | 0.98 | 0.003 | NM_012316 | ENSG00000025800 | KPNA6 | karyopherin alpha 6 (importin alpha 7) |
| 1.92 | 0.75 | 0.39 | -1.36 | 0.003 | AL117623 | ENSG00000108306 | FBXL20 | hypothetical protein LOC90110 |
| 0.70 | 1.78 | 2.52 | 1.34 | 0.004 | AF161411 | ENSG00000148153 | INIP | chromosome 9 open reading frame 80 |
| 0.81 | 1.71 | 2.10 | 1.07 | 0.004 | AK023251 | ENSG00000233237 | LINC00472 | chromosome 6 open reading frame 155 |
| 0.64 | 1.62 | 2.53 | 1.34 | 0.004 | AK025259 | ENSG00000266714 | MYO15B | myosin XVB pseudogene |
| 0.51 | 1.08 | 2.12 | 1.08 | 0.004 | NM_004781 | ENSG00000049245 | VAMP3 | vesicle-associated membrane protein 3 (cellubrevin |
| 1.38 | 0.61 | 0.44 | -1.19 | 0.004 | NM_017590 | ENSG00000100403 | ZC3H7B | zinc finger CCCH-type containing 7B |
| 1.45 | 0.75 | 0.51 | -0.96 | 0.004 | AL137554 | ENSG00000102543 | CDADC1 | cytidine and dCMP deaminase domain containing 1 |
| 1.83 | 0.89 | 0.49 | -1.04 | 0.004 | AK026745 | ENSG00000111554 | MDM1 | Mdm1 nuclear protein homolog (mouse) |
| 0.57 | 1.30 | 2.30 | 1.20 | 0.004 | NM_002738 | ENSG00000166501 | PRKCB | protein kinase C. beta 1 |
| 0.85 | 1.63 | 1.91 | 0.94 | 0.004 | NM_014624 | ENSG00000197956 | S100A6 | S100 calcium binding protein A6 |
| 0.74 | 1.42 | 1.93 | 0.95 | 0.005 | NM_017925 | ENSG00000137145 | DENND4C | DENN/MADD domain containing 4C |
| 1.53 | 0.77 | 0.50 | -0.99 | 0.005 | NM_001917 | ENSG00000110887 | DAO | D-amino-acid oxidase |
| 0.58 | 1.18 | 2.05 | 1.03 | 0.005 | NM_018647 | ENSG00000127863 | TNFRSF19 | tumor necrosis factor receptor superfamily. member 19 |
| 0.91 | 1.87 | 2.06 | 1.04 | 0.005 | AF086231 | ENSG00000186951 | PPARA | peroxisome proliferator-activated receptor alpha |
| 0.90 | 1.71 | 1.91 | 0.93 | 0.005 | NM_019082 | ENSG00000136271 | DDX56 | DEAD (Asp-Glu-Ala-Asp) box polypeptide 56 |
| 0.49 | 1.10 | 2.27 | 1.18 | 0.005 | NM_003633 | ENSG00000171617 | ENC1 | ectodermal-neural cortex (with BTB-like domain) |
| 1.38 | 0.74 | 0.53 | -0.90 | 0.005 | NM_005461 | ENSG00000204103 | MAFB | v-maf musculoaponeurotic fibrosarcoma oncogene homolog B (avian) |
| 0.67 | 1.44 | 2.14 | 1.10 | 0.005 | U66050 | ENSG00000197620 | CXorf40A | chromosome X open reading frame 40A |
| 0.83 | 1.74 | 2.10 | 1.07 | 0.005 | D56495 | ENSG00000204787 | REG1CP | regenerating islet-derived 1 pseudogene |
| 0.67 | 1.78 | 2.65 | 1.41 | 0.005 | AK024144 | #N/A | #N/A |  |
| 0.86 | 1.70 | 1.99 | 0.99 | 0.006 | NM_000392 | ENSG00000023839 | ABCC2 | ATP-binding cassette. sub-family C (CFTR/MRP). member 2 |
| 1.31 | 0.62 | 0.48 | -1.07 | 0.006 | AF071554 | ENSG00000171956 | FOXB1 | forkhead box B1 |
| 1.85 | 0.79 | 0.43 | -1.22 | 0.006 | NM_002454 | ENSG00000124275 | MTRR | 5-methyltetrahydrofolate-homocysteine methyltransferase reductase |
| 1.76 | 0.66 | 0.37 | -1.43 | 0.006 | AK024181 | #N/A | #N/A | hypothetical protein LOC742340 |
| 0.73 | 1.34 | 1.82 | 0.87 | 0.006 | AL110227 | ENSG00000088256 | GNA11 | guanine nucleotide binding protein (G protein). alpha 11 (Gq class) |
| 2.43 | 0.83 | 0.34 | -1.56 | 0.007 | NM_002588 | ENSG00000240184 | PCDHGC3 | protocadherin gamma subfamily C. 3 |
| 1.30 | 0.71 | 0.54 | -0.88 | 0.007 | AF007192 | ENSG00000169876 | MUC17 | mucin 17, cell surface associated |
| 0.65 | 1.17 | 1.80 | 0.85 | 0.007 | NM_014565 | ENSG00000172146 | OR1A1 | olfactory receptor. family 1. subfamily A. member 1 |
| 0.56 | 1.25 | 2.24 | 1.16 | 0.007 | NM_015959 | ENSG00000213593 | TMX2 | thioredoxin domain containing 14 |
| 0.75 | 1.35 | 1.80 | 0.85 | 0.008 | AF111849 | ENSG00000012660 | ELOVL5 | ELOVL family member 5. elongation of long chain fatty acids (FEN1/Elo2. SUR4/Elo3-like. yeast) |
| 0.59 | 1.22 | 2.08 | 1.05 | 0.008 | NM_018025 | ENSG00000076650 | GPATCH1 | G patch domain containing 1 |
| 1.29 | 0.73 | 0.57 | -0.82 | 0.009 | AL133069 | ENSG00000134452 | FBXO18 | F-box protein. helicase. 18 |
| 0.88 | 1.59 | 1.80 | 0.85 | 0.009 | AK025657 | ENSG00000115084 | SLC35F5 | solute carrier family 35. member F5 |
| 1.56 | 0.90 | 0.58 | -0.80 | 0.009 | NM_014495 | ENSG00000132855 | ANGPTL3 | angiopoietin-like 3 |
| 1.88 | 0.83 | 0.44 | -1.18 | 0.010 | AF085877 | ENSG00000260233 | SSSCA1-AS1 | SSSCA1 antisense RNA 1 (head to head) |
| 0.86 | 1.53 | 1.79 | 0.84 | 0.010 | U49349 | ENSG00000145675 | PIK3R1 | phosphoinositide-3-kinase. regulatory subunit 1 (alpha) |
| 1.04 | 0.60 | 0.57 | -0.80 | 0.010 | AF055030 | ENSG00000185127 | C6orf120 | chromosome 6 open reading frame 120 |
| 0.69 | 1.42 | 2.07 | 1.05 | 0.011 | NM_014521 | ENSG00000130147 | SH3BP4 | SH3-domain binding protein 4 |
| 1.49 | 0.71 | 0.48 | -1.07 | 0.011 | NM_000539 | ENSG00000163914 | RHO | rhodopsin |
| 1.38 | 0.81 | 0.58 | -0.78 | 0.011 | NM_001943 | ENSG00000046604 | DSG2 | desmoglein 2 |
| 0.90 | 1.56 | 1.72 | 0.79 | 0.011 | NM_002276 | ENSG00000171345 | KRT19 | keratin 19 |
| 0.75 | 1.77 | 2.38 | 1.25 | 0.011 | AK022790 | ENSG00000139641 | ESYT1 | family with sequence similarity 62 (C2 domain containing). member A |
| 1.81 | 0.83 | 0.46 | -1.12 | 0.012 | AB040929 | ENSG00000113805 | CNTN3 | contactin 3 (plasmacytoma associated) |
| 0.71 | 1.23 | 1.73 | 0.79 | 0.012 | NM_004167 | ENSG00000275718 | CCL15 | chemokine (C-C motif) ligand 15 |
| 1.47 | 0.73 | 0.50 | -1.01 | 0.012 | AB023166 | ENSG00000122966 | CIT | citron |
| 0.81 | 1.43 | 1.75 | 0.81 | 0.012 | AK025322 | ENSG00000132623 | ANKEF1 | ankyrin repeat domain 5 |
| 0.79 | 1.58 | 2.00 | 1.00 | 0.012 | AF069469 | #N/A | #N/A | sterol-C5-desaturase |
| 0.74 | 1.42 | 1.93 | 0.95 | 0.012 | NM_002753 | ENSG00000109339 | MAPK10 | mitogen-activated protein kinase 10 |
| 0.70 | 1.35 | 1.92 | 0.94 | 0.012 | AF264627 | #N/A | #N/A | uncharacterized gastric protein YC12P |
| 1.28 | 0.60 | 0.47 | -1.09 | 0.012 | AB033053 | ENSG00000173276 | ZBTB21 | zinc finger protein 295 |
| 1.55 | 0.91 | 0.59 | -0.77 | 0.012 | AK025587 | ENSG00000135220 | UGT2A3 | UDP glucuronosyltransferase 2 family. polypeptide A3 |
| 0.74 | 1.27 | 1.71 | 0.77 | 0.012 | AB020706 | ENSG00000183020 | AP2A2 | adaptor-related protein complex 2. alpha 2 subunit |
| 1.34 | 0.80 | 0.60 | -0.75 | 0.013 | AF007143 | ENSG00000140557 | ST8SIA2 | ST8 alpha-N-acetyl-neuraminide alpha-2,8-sialyltransferase 2 |
| 0.67 | 1.32 | 1.95 | 0.97 | 0.013 | NM_002018 | ENSG00000177731 | FLII | flightless I homolog (Drosophila) |
| 1.24 | 0.65 | 0.53 | -0.93 | 0.014 | AL133561 | #N/A | #N/A | DKFZP434B061 protein |
| 0.70 | 1.24 | 1.77 | 0.83 | 0.014 | AL137437 | ENSG00000197106 | SLC6A17 | solute carrier family 6. member 17 |
| 0.68 | 1.16 | 1.69 | 0.76 | 0.015 | AK025905 | ENSG00000164736 | SOX17 | SRY (sex determining region Y)-box 17 |
| 0.65 | 1.18 | 1.81 | 0.85 | 0.015 | NM_018699 | ENSG00000138738 | PRDM5 | PR domain containing 5 |
| 1.18 | 0.65 | 0.55 | -0.87 | 0.015 | AJ227875 | #N/A | #N/A |  |
| 1.09 | 0.63 | 0.58 | -0.79 | 0.015 | NM_002642 | ENSG00000135845 | PIGC | phosphatidylinositol glycan anchor biosynthesis. class C |
| 1.47 | 0.86 | 0.58 | -0.78 | 0.015 | AB033065 | ENSG00000174145 | NWD2 | NACHT and WD repeat domain containing 2 |
| 1.25 | 0.70 | 0.56 | -0.83 | 0.016 | Y11177 | ENSG00000122691 | TWIST1 | twist homolog 1 |
| 1.27 | 0.72 | 0.57 | -0.82 | 0.016 | AL049300 | ENSG00000197565 | COL4A6 | collagen type IV alpha 6 chain |
| 0.69 | 1.15 | 1.67 | 0.74 | 0.016 | NM_001304 | ENSG00000108582 | CPD | carboxypeptidase D |
| 0.79 | 1.44 | 1.82 | 0.86 | 0.016 | AK026497 | ENSG00000100811 | YY1 | YY1 transcription factor |
| 1.18 | 0.71 | 0.60 | -0.74 | 0.016 | AB002298 | ENSG00000133401 | PDZD2 | PDZ domain containing 2 |
| 1.30 | 0.76 | 0.58 | -0.77 | 0.017 | NM_016945 | ENSG00000128519 | TAS2R16 | taste receptor. type 2. member 16 |
| 0.98 | 1.63 | 1.67 | 0.74 | 0.017 | NM_000224 | #N/A | #N/A | keratin 18 |
| 1.05 | 0.65 | 0.61 | -0.70 | 0.017 | NM_016357 | ENSG00000050405 | LIMA1 | LIM domain and actin binding 1 |
| 1.38 | 0.82 | 0.59 | -0.75 | 0.017 | NM_002902 | ENSG00000117906 | RCN2 | eticulocalbin 2. EF-hand calcium binding domain |
| 1.37 | 0.69 | 0.50 | -1.00 | 0.018 | NM_002451 | ENSG00000099810 | MTAP | methylthioadenosine phosphorylase |
| 1.37 | 0.55 | 0.40 | -1.32 | 0.018 | AF089088 | ENSG00000142330 | CAPN10 | calpain 10 |
| 0.54 | 1.19 | 2.20 | 1.14 | 0.018 | AL157425 | ENSG00000242808 | SOX2-OT | SOX2 overlapping transcript (non-protein coding) |
| 0.61 | 1.30 | 2.14 | 1.10 | 0.018 | AL359562 | ENSG00000185619 | PCGF3 | polycomb group ring finger 3 |
| 1.34 | 0.74 | 0.55 | -0.86 | 0.018 | NM_013293 | ENSG00000164548 | TRA2A | transformer-2 alpha |
| 0.53 | 1.18 | 2.23 | 1.16 | 0.018 | S68954 | ENSG00000125144 | MT1G | metallothionein 1G |
| 1.50 | 0.75 | 0.50 | -1.00 | 0.018 | NM_005993 | ENSG00000141556 | TBCD | tubulin folding cofactor D |
| 0.66 | 1.32 | 2.01 | 1.01 | 0.018 | AB029025 | ENSG00000064042 | LIMCH1 | LIM and calponin homology domains 1 |
| 0.63 | 1.20 | 1.91 | 0.93 | 0.019 | AF090887 | ENSG00000177853 | ZNF518A | zinc finger protein 518A |
| 1.25 | 0.77 | 0.62 | -0.69 | 0.019 | AF086434 | ENSG00000121964 | GTDC1 | glycosyltransferase like domain containing 1 |
| 0.86 | 1.39 | 1.62 | 0.70 | 0.019 | AF251189 | #N/A | #N/A |  |
| 1.11 | 0.70 | 0.62 | -0.68 | 0.019 | AF130071 | #N/A | #N/A |  |
| 1.55 | 0.96 | 0.62 | -0.69 | 0.020 | NM_002623 | ENSG00000101132 | PFDN4 | prefoldin subunit 4 |
| 1.07 | 0.66 | 0.62 | -0.69 | 0.020 | NM_000343 | ENSG00000100170 | SLC5A1 | solute carrier family 5 (sodium/glucose cotransporter). member 1 |
| 1.51 | 0.95 | 0.63 | -0.68 | 0.020 | NM_014353 | ENSG00000167964 | RAB26 | RAB26, member RAS oncogene family |
| 1.39 | 0.82 | 0.59 | -0.77 | 0.020 | NM_005382 | ENSG00000104722 | NEFM | neurofilament. medium polypeptide 150kDa |
| 0.67 | 1.38 | 2.06 | 1.04 | 0.020 | AL049301 | #N/A | #N/A |  |
| 1.39 | 0.85 | 0.61 | -0.70 | 0.021 | AK022292 | ENSG00000113761 | ZNF346 | zinc finger protein 346 |
| 1.10 | 0.57 | 0.52 | -0.95 | 0.021 | AK025126 | ENSG00000171132 | PRKCE | protein kinase C epsilon |
| 0.73 | 1.53 | 2.08 | 1.06 | 0.021 | NM_014700 | ENSG00000090565 | RAB11FIP3 | RAB11 family interacting protein 3 (class II) |
| 1.37 | 0.83 | 0.60 | -0.73 | 0.021 | AJ243222 | ENSG00000200785 | SNORD8 | small nucleolar RNA. C/D box 8 |
| 0.83 | 1.58 | 1.90 | 0.93 | 0.021 | AL355741 | ENSG00000270093 | #N/A |  |
| 1.51 | 0.83 | 0.55 | -0.86 | 0.021 | NM_007136 | ENSG00000174255 | ZNF80 | zinc finger protein 80 |
| 1.66 | 0.81 | 0.49 | -1.04 | 0.022 | NM_001546 | ENSG00000172201 | ID4 | inhibitor of DNA binding 4. dominant negative helix-loop-helix protein |
| 0.69 | 1.22 | 1.78 | 0.83 | 0.022 | NM_004248 | ENSG00000119973 | PRLHR | prolactin releasing hormone receptor |
| 0.86 | 2.23 | 2.58 | 1.37 | 0.022 | NM_020128 | #N/A | #N/A | Mdm1 nuclear protein homolog (mouse) |
| 1.16 | 0.72 | 0.62 | -0.70 | 0.023 | AK027246 | ENSG00000109929 | SC5D | sterol-C5-desaturase |
| 1.17 | 0.51 | 0.44 | -1.19 | 0.023 | AK024507 | ENSG00000144596 | GRIP2 | glutamate receptor interacting protein 2 |
| 1.14 | 0.69 | 0.60 | -0.74 | 0.023 | NM_004157 | ENSG00000114302 | PRKAR2A | protein kinase. cAMP-dependent. regulatory. type II. alpha |
| 1.39 | 0.73 | 0.53 | -0.92 | 0.024 | NM_006344 | ENSG00000132514 | CLEC10A | C-type lectin domain family 10. member A |
| 1.02 | 0.57 | 0.56 | -0.83 | 0.024 | NM_015340 | ENSG00000011376 | LARS2 | leucyl-tRNA synthetase 2. mitochondrial |
| 0.90 | 1.44 | 1.61 | 0.69 | 0.024 | NM_005842 | ENSG00000136158 | SPRY2 | sprouty homolog 2 (Drosophila) |
| 1.34 | 0.86 | 0.64 | -0.65 | 0.024 | NM_019109 | ENSG00000033011 | ALG1 | asparagine-linked glycosylation 1 homolog |
| 0.90 | 1.67 | 1.87 | 0.90 | 0.025 | NM_012079 | ENSG00000185000 | DGAT1 | diacylglycerol O-acyltransferase homolog 1 (mouse) |
| 1.34 | 0.76 | 0.57 | -0.82 | 0.025 | AF268872 | ENSG00000132394 | EEFSEC | eukaryotic elongation factor. selenocysteine-tRNA-specific |
| 1.39 | 0.82 | 0.59 | -0.76 | 0.025 | NM_016070 | ENSG00000181610 | MRPS23 | mitochondrial ribosomal protein S23 |
| 0.65 | 1.19 | 1.83 | 0.88 | 0.025 | NM_012345 | ENSG00000083635 | NUFIP1 | nuclear fragile X mental retardation protein interacting protein 1 |
| 1.41 | 0.84 | 0.60 | -0.74 | 0.027 | NM_006758 | ENSG00000160201 | U2AF1 | U2 small nuclear RNA auxiliary factor 1 |
| 1.41 | 0.88 | 0.62 | -0.68 | 0.027 | NM_006245 | ENSG00000112640 | PPP2R5D | protein phosphatase 2. regulatory subunit B'. delta isoform |
| 1.78 | 0.87 | 0.49 | -1.03 | 0.027 | NM_000071 | ENSG00000111057 | KRT18 | keratin 18 |
| 1.44 | 0.77 | 0.53 | -0.91 | 0.027 | NM_018664 | ENSG00000123685 | BATF3 | basic leucine zipper transcription factor. ATF-like 3 |
| 0.75 | 1.24 | 1.64 | 0.72 | 0.027 | AK023734 | ENSG00000163293 | NIPAL1 | similar to non-imprinted in Prader-Willi/Angelman syndrome 2 |
| 1.38 | 0.87 | 0.63 | -0.67 | 0.027 | AK023680 | ENSG00000158615 | PPP1R15B |  |
| 1.36 | 0.77 | 0.56 | -0.83 | 0.028 | AK022451 | ENSG00000081760 | AACS | acetoacetyl-CoA synthetase |
| 0.80 | 1.41 | 1.77 | 0.82 | 0.028 | NM_004595 | ENSG00000102172 | SMS | spermine synthase |
| 1.44 | 0.93 | 0.64 | -0.63 | 0.028 | NM_017692 | ENSG00000137074 | APTX | aprataxin |
| 1.31 | 0.79 | 0.60 | -0.74 | 0.028 | NM_018342 | ENSG00000164124 | TMEM144 | transmembrane protein 144 |
| 1.40 | 0.75 | 0.54 | -0.89 | 0.028 | NM_020528 | ENSG00000183570 | PCBP3 | poly(rC) binding protein 3 |
| 0.81 | 1.46 | 1.81 | 0.85 | 0.029 | AL110245 | ENSG00000137266 | SLC22A23 | solute carrier family 22. member 23 |
| 1.21 | 0.77 | 0.63 | -0.66 | 0.029 | NM_002066 | ENSG00000104499 | GML | glycosylphosphatidylinositol anchored molecule like protein |
| 1.41 | 0.77 | 0.55 | -0.87 | 0.029 | AK025786 | ENSG00000234380 | LINC01426 |  |
| 0.70 | 1.13 | 1.61 | 0.69 | 0.030 | NM_018728 | ENSG00000128833 | MYO5C | myosin VC |
| 0.83 | 1.57 | 1.89 | 0.92 | 0.030 | NM_005682 | ENSG00000205336 | ADGRG1 | G protein-coupled receptor 56 |
| 1.40 | 0.90 | 0.64 | -0.63 | 0.031 | S56365 | ENSG00000134982 | APC | APC, WNT signaling pathway regulator |
| 1.54 | 0.87 | 0.56 | -0.83 | 0.031 | U96191 | #N/A | #N/A |  |
| 0.86 | 1.35 | 1.58 | 0.66 | 0.031 | NM_005623 | ENSG00000108700 | CCL8 | chemokine (C-C motif) ligand 8 |
| 0.70 | 1.23 | 1.76 | 0.82 | 0.031 | NM_012464 | ENSG00000038295 | TLL1 | tolloid-like 1 |
| 1.31 | 0.79 | 0.60 | -0.73 | 0.031 | AF086073 | ENSG00000149294 | NCAM1 | neural cell adhesion molecule 1 |
| 0.71 | 1.34 | 1.89 | 0.92 | 0.032 | AK025719 | ENSG00000167244 | IGF2 | insulin-like growth factor 2 |
| 0.87 | 1.32 | 1.52 | 0.60 | 0.032 | AL137364 | ENSG00000170456 | DENND5B | hypothetical protein MGC24039 |
| 0.56 | 1.12 | 2.02 | 1.01 | 0.032 | NM_016531 | ENSG00000109787 | KLF3 | Kruppel-like factor 3 (basic) |
| 1.60 | 0.89 | 0.56 | -0.84 | 0.032 | NM_004548 | ENSG00000140990 | NDUFB10 | NADH dehydrogenase (ubiquinone) 1 beta subcomplex. 10. 22kDa |
| 1.24 | 0.80 | 0.65 | -0.63 | 0.033 | NM_005922 | ENSG00000085511 | MAP3K4 | mitogen-activated protein kinase kinase kinase 4 |
| 1.28 | 0.77 | 0.60 | -0.74 | 0.033 | NM_005221 | ENSG00000105880 | DLX5 | distal-less homeobox 5 |
| 0.82 | 1.41 | 1.73 | 0.79 | 0.034 | NM_003488 | ENSG00000121057 | AKAP1 | A kinase (PRKA) anchor protein 1 |
| 1.48 | 0.79 | 0.53 | -0.91 | 0.034 | AK026219 | #N/A | #N/A |  |
| 1.04 | 0.65 | 0.62 | -0.69 | 0.034 | NM_004599 | ENSG00000198911 | SREBF2 | sterol regulatory element binding transcription factor 2 |
| 0.72 | 1.26 | 1.74 | 0.80 | 0.034 | NM_004870 | ENSG00000129255 | MPDU1 | mannose-P-dolichol utilization defect 1 |
| 1.16 | 0.74 | 0.64 | -0.65 | 0.034 | NM_018148 | ENSG00000140471 | LINS1 | lines homolog 1 (Drosophila) |
| 0.90 | 1.47 | 1.63 | 0.70 | 0.035 | U62822 | ENSG00000264229 | RNU4ATAC | RNA, U4atac small nuclear (U12-dependent splicing) |
| 1.44 | 0.95 | 0.66 | -0.60 | 0.035 | NM_016175 | ENSG00000161010 | MRNIP | MRN complex interacting protein |
| 0.83 | 1.25 | 1.51 | 0.60 | 0.036 | X90978 | ENSG00000159216 | RUNX1 | runt-related transcription factor 1 (acute myeloid leukemia 1; aml1 oncogene) |
| 0.67 | 1.09 | 1.62 | 0.69 | 0.036 | NM_014958 | ENSG00000198844 | ARHGEF15 | Rho guanine nucleotide exchange factor 15 |
| 0.81 | 2.18 | 2.69 | 1.43 | 0.036 | NM_004580 | ENSG00000069974 | RAB27A | RAB27A. member RAS oncogene family |
| 1.08 | 0.71 | 0.65 | -0.61 | 0.037 | AB040889 | ENSG00000250305 | KIAA1456 | chromosome 8 open reading frame 79 |
| 0.69 | 1.04 | 1.50 | 0.58 | 0.038 | AF052172 | #N/A | #N/A |  |
| 0.79 | 1.21 | 1.54 | 0.62 | 0.039 | NM_000377 | ENSG00000015285 | WAS | Wiskott-Aldrich syndrome (eczema-thrombocytopenia) |
| 1.58 | 0.94 | 0.60 | -0.75 | 0.040 | NM_014371 | ENSG00000011243 | AKAP8L | A kinase (PRKA) anchor protein 8-like |
| 1.50 | 0.87 | 0.58 | -0.78 | 0.040 | AK027073 | ENSG00000171017 | LRRC8E | leucine rich repeat containing 8 family. member E |
| 1.93 | 0.79 | 0.41 | -1.29 | 0.041 | NM_006030 | ENSG00000007402 | CACNA2D2 | calcium channel. voltage-dependent. alpha 2/delta subunit 2 |
| 0.76 | 1.33 | 1.76 | 0.82 | 0.041 | AK022878 | ENSG00000100439 | ABHD4 | abhydrolase domain containing 4 |
| 1.34 | 0.84 | 0.63 | -0.67 | 0.042 | AK001126 | ENSG00000181135 | ZNF707 | zinc finger protein 707 |
| 0.91 | 1.56 | 1.72 | 0.78 | 0.042 | NM_018278 | ENSG00000186638 | KIF24 | kinesin family member 24 |
| 1.25 | 0.82 | 0.66 | -0.60 | 0.042 | AK000930 | ENSG00000106617 | PRKAG2 | protein kinase AMP-activated non-catalytic subunit gamma 2 |
| 0.82 | 1.27 | 1.55 | 0.63 | 0.044 | AB007964 | ENSG00000227372 | TP73-AS1 | TP73 antisense RNA 1 |
| 1.23 | 0.74 | 0.60 | -0.74 | 0.045 | X76978 | ENSG00000235608 | NKX1-1 | NK1 homeobox 1 |
| 1.13 | 0.68 | 0.60 | -0.73 | 0.047 | AF009308 | ENSG00000112902 | SEMA5A | semaphorin 5A |
| 1.30 | 0.82 | 0.63 | -0.66 | 0.047 | AB033028 | ENSG00000158352 | SHROOM4 | shroom family member 4 |
| 1.25 | 0.85 | 0.68 | -0.55 | 0.048 | NM_014454 | ENSG00000080546 | SESN1 | sestrin 1 |
| 1.35 | 0.85 | 0.63 | -0.67 | 0.048 | AK025363 | #N/A | #N/A |  |
| 0.83 | 1.26 | 1.52 | 0.60 | 0.048 | AL080135 | ENSG00000233864 | TTTY15 | testis-specific transcript. Y-linked 15 |
| 1.10 | 0.72 | 0.66 | -0.60 | 0.050 | NM_000720 | ENSG00000157388 | CACNA1D | calcium channel. voltage-dependent. L type. alpha 1D subunit |
