# Supplementary material for: Clinical relevance of the transcriptional signature regulated by CDC42 in colorectal cancer
Source: Oncotarget. 2017 Mar 1;8(16):26755–70. doi: 10.18632/oncotarget.15815 (PMC5432295; doi:10.18632/oncotarget.15815)
Supplement: Supplementary file 3 [file oncotarget-08-26755-s003.docx]

**Table S3: Full table of Gene Set Enrichment Analyses for curated gene sets/ canonical pathways (p-value < 0.05) for CDC42 regulated genes in SW620 cells**

| Term | Count | Fold enrichment | PValue | Genes |
| --- | --- | --- | --- | --- |
| BLALOCK_ALZHEIMERS_DISEASE_UP | 29 | 1.64 | 0.005 | AKAP1,AKAP8L,APTX,ARHGEF15,DAO,FLII,HLA-DPA1,HSPB2,ID4,KPNA6,MAFB,MDM1,MYO15B,MYO5C,NCAM1,NEK1,NUFIP1,PPP2R5D,PRKAR2A,RBM6,RPS6KA1,SREBF2,TAS2R16,TLL1,VAMP3,WAS,YY1,ZBTB20,ZC3H7B |
| BENPORATH_ES_WITH_H3K27ME3 | 19 | 2.08 | 0.002 | BATF3,CACNA1D,COL4A6,DLX5,FOXB1,HEY1,MAFB,NCAM1,NEFM,PDZD2,PRKAG2,PRKCB,PRKCE,PRLHR,RAB11FIP3,SOX17,ST8SIA2,TLL1,TWIST1 |
| BENPORATH_EED_TARGETS | 18 | 2.03 | 0.003 | BATF3,CACNA1D,COL4A6,DLX5,FOXB1,HEY1,IGF2,MAFB,NCAM1,NEFM,PDZD2,PRKCB,PRKCE,PRLHR,SOX17,ST8SIA2,TLL1,TWIST1 |
| BENPORATH_SUZ12_TARGETS | 15 | 1.75 | 0.023 | BATF3,CACNA1D,COL4A6,FOXB1,HEY1,IGF2,MAFB,NCAM1,NEFM,PDZD2,PRKCE,PRLHR,SOX17,ST8SIA2,TLL1 |
| BENPORATH_PRC2_TARGETS | 14 | 2.55 | 0.001 | BATF3,CACNA1D,COL4A6,FOXB1,HEY1,MAFB,NCAM1,NEFM,PDZD2,PRKCE,PRLHR,SOX17,ST8SIA2,TLL1 |
| CREIGHTON_ENDOCRINE_THERAPY_RESISTANCE_3 | 13 | 1.84 | 0.024 | ALG1,CDADC1,CPD,ENC1,HEY1,LIMCH1,MAPK10,PPARA,RAB26,RAB27A,RPS6KA1,S100A6,TNFRSF19 |
| WONG_ADULT_TISSUE_STEM_MODULE | 13 | 1.82 | 0.026 | CACNA2D2,ELOVL5,ID4,LIMCH1,MYO5C,NCAM1,PRDM5,PRKCB,PRKCE,RAB27A,S100A6,SESN1,ZBTB20 |
| WAKABAYASHI_ADIPOGENESIS_PPARG_BOUND_8D | 12 | 1.99 | 0.017 | AACS,AKAP1,APTX,CDADC1,DLX5,FBXO18,KRT19,MPDU1,NDUFB10,PPP2R5D,PRKAG2,PRKCE |
| MILI_PSEUDOPODIA_HAPTOTAXIS_DN | 12 | 1.98 | 0.018 | AKAP1,AP2A2,C6orf120,CPD,ELOVL5,ENC1,ESYT1,MPDU1,NCAM1,PPP1R15B,SLC22A23,SREBF2 |
| IVANOVA_HEMATOPOIESIS_STEM_CELL_AND_PROGENITOR | 11 | 1.82 | 0.039 | CAPN10,FOXB1,KRT18,NUFIP1,PRDM5,PRKAG2,PRKCE,RAB26,RCN2,SESN1,SMS |
| BOQUEST_STEM_CELL_CULTURED_VS_FRESH_UP | 10 | 2.42 | 0.008 | CBS,CCL8,ELOVL5,HLA-DPA1,KRT18,LIMCH1,MAFB,PIK3R1,SPRY2,ZBTB20 |
| MATSUDA_NATURAL_KILLER_DIFFERENTIATION | 10 | 2.12 | 0.020 | CPD,DSG2,ENC1,HEY1,ID4,MAPK10,NDUFB10,RBM6,SESN1,SLC35F5 |
| BONOME_OVARIAN_CANCER_SURVIVAL_SUBOPTIMAL_DEBULKING | 10 | 1.88 | 0.040 | APC,LIMCH1,LRRC8E,MTAP,PCGF3,PIK3R1,SESN1,THSD7A,WAS,ZNF518A |
| BLALOCK_ALZHEIMERS_DISEASE_INCIPIENT_UP | 9 | 2.21 | 0.020 | ARHGEF15,DAO,FLII,HLA-DPA1,ID4,MDM1,PRKAR2A,WAS,ZC3H7B |
| SWEET_LUNG_CANCER_KRAS_DN | 9 | 2.08 | 0.029 | DENND4C,HEY1,LIMCH1,PRKCE,SESN1,SOX17,TNFRSF19,VAMP3,ZBTB20 |
| ZHOU_INFLAMMATORY_RESPONSE_LIVE_UP | 9 | 2.07 | 0.030 | AKAP1,HEY1,ID4,KRT19,NIPAL1,SEMA6A,SLC22A23,SLC5A1,ZC3H7B |
| DELYS_THYROID_CANCER_UP | 9 | 1.97 | 0.040 | ADORA1,CPD,ENC1,FAP,FLII,KRT19,RAB27A,RCN2,RUNX1 |
| MILI_PSEUDOPODIA_CHEMOTAXIS_DN | 9 | 1.90 | 0.048 | AKAP1,ALG1,AP2A2,COL4A6,DGAT1,ESYT1,KLF3,SREBF2,TRA2A |
| BOQUEST_STEM_CELL_DN | 8 | 3.34 | 0.003 | ARHGEF15,DSG2,HLA-DPA1,MYO5C,SEMA6A,SOX17,THSD7A,ZNF385D |
| FARMER_BREAST_CANCER_APOCRINE_VS_BASAL | 8 | 2.40 | 0.018 | ELOVL5,KRT18,LIMA1,LIMCH1,PFDN4,PIK3R1,S100A6,SH3BP4 |
| MIKKELSEN_MCV6_HCP_WITH_H3K27ME3 | 8 | 2.33 | 0.021 | BATF3,CACNA1D,CACNA2D2,MAFB,NRG3,PFDN4,PRLHR,SLC6A17 |
| MCBRYAN_PUBERTAL_BREAST_3_4WK_UP | 7 | 4.52 | 0.001 | ID4,KRT18,KRT19,LIMA1,MAFB,PRKCE,RUNX1 |
| WANG_SMARCE1_TARGETS_UP | 7 | 2.79 | 0.013 | DSG2,FAP,KRT18,LIMA1,MAFB,RAB27A,TNFRSF19 |
| GABRIELY_MIR21_TARGETS | 7 | 2.28 | 0.034 | APC,LIMCH1,NEK1,PIK3R1,PPARA,SESN1,ZBTB20 |
| LABBE_WNT3A_TARGETS_DN | 6 | 6.90 | 0.000 | DGAT1,GML,HEY1,PRKCB,S100A6,SEMA5A |
| WU_CELL_MIGRATION | 6 | 3.65 | 0.006 | KRT18,KRT19,LIMA1,LIMCH1,MT1G,S100A6 |
| THUM_SYSTOLIC_HEART_FAILURE_DN | 6 | 2.69 | 0.024 | AKAP8L,NEK1,PDZD2,RBM6,TRA2A,ZBTB20 |
| VERHAAK_AML_WITH_NPM1_MUTATED_DN | 6 | 2.37 | 0.041 | CACNA2D2,CLEC10A,HLA-DPA1,NCAM1,SH3BP4,THSD7A |
| MIYAGAWA_TARGETS_OF_EWSR1_ETS_FUSIONS_UP | 6 | 2.37 | 0.041 | HSPB2,KLF3,KRT18,KRT19,MAFB,PPARA |
| HOSHIDA_LIVER_CANCER_SUBCLASS_S2 | 5 | 4.53 | 0.005 | CPD,IGF2,KLF3,PIGC,SREBF2 |
| GOZGIT_ESR1_TARGETS_UP | 5 | 3.43 | 0.015 | CACNA1D,LIMCH1,PRKAG2,SEMA5A,THSD7A |
| GAUSSMANN_MLL_AF4_FUSION_TARGETS_A_UP | 5 | 2.88 | 0.029 | APC,KIF24,NCAM1,NIPAL1,ZNF518A |
| SHEPARD_CRUSH_AND_BURN_MUTANT_DN | 5 | 2.80 | 0.033 | FOXB1,HEY1,PCDHGC3,RHO,ST8SIA2 |
| DELYS_THYROID_CANCER_DN | 5 | 2.66 | 0.039 | ID4,IGF2,MDM1,MT1G,NCAM1 |
| PEREZ_TP53_AND_TP63_TARGETS | 5 | 2.63 | 0.041 | GNA11,ID4,MAFB,RAB11FIP3,ST8SIA2 |
| DAWSON_METHYLATED_IN_LYMPHOMA_TCL1 | 4 | 6.55 | 0.003 | ID4,NCAM1,SOX17,SPRY2 |
| BHATTACHARYA_EMBRYONIC_STEM_CELL | 4 | 5.01 | 0.008 | DSG2,KRT18,SEMA6A,SMS |
| CHANG_IMMORTALIZED_BY_HPV31_UP | 4 | 5.01 | 0.008 | APC,HEY1,MAP3K4,SOX17 |
| SHETH_LIVER_CANCER_VS_TXNIP_LOSS_PAM5 | 4 | 4.60 | 0.011 | CIT,NCAM1,RUNX1,SEMA5A |
| GRABARCZYK_BCL11B_TARGETS_UP | 4 | 4.48 | 0.012 | PIK3R1,RAB27A,SLC35F5,U2AF1 |
| NELSON_RESPONSE_TO_ANDROGEN_UP | 4 | 4.26 | 0.014 | CPD,ELOVL5,KRT19,SMS |
| GRAHAM_CML_DIVIDING_VS_NORMAL_QUIESCENT_DN | 4 | 3.78 | 0.021 | HLA-DPA1,LIMCH1,MYO5C,TRA2A |
| LABBE_TARGETS_OF_TGFB1_AND_WNT3A_DN | 4 | 3.70 | 0.022 | DSG2,KRT19,MAPK10,S100A6 |
| CHIARADONNA_NEOPLASTIC_TRANSFORMATION_KRAS_UP | 4 | 3.41 | 0.029 | NCAM1,PRKCB,RRM1,SMS |
| MEISSNER_NPC_HCP_WITH_H3K4ME3_AND_H3K27ME3 | 4 | 3.41 | 0.029 | ADORA1,FOXB1,NEFM,ST8SIA2 |
| SERVITJA_LIVER_HNF1A_TARGETS_UP | 4 | 3.28 | 0.033 | ADORA1,ENC1,RAB27A,TNFRSF19 |
| REN_ALVEOLAR_RHABDOMYOSARCOMA_UP | 4 | 3.21 | 0.035 | CACNA2D2,HEY1,NCAM1,SEMA6A |
| WIERENGA_STAT5A_TARGETS_GROUP1 | 4 | 3.15 | 0.037 | BATF3,CBS,ENC1,SH3BP4 |
| JOHNSTONE_PARVB_TARGETS_3_DN | 4 | 0.43 | 0.040 | MDM1,PRKAR2A,RRM1,TRA2A |
| WHITFIELD_CELL_CYCLE_G1_S | 4 | 2.94 | 0.047 | FBXL20,MDM1,RUNX1,TRA2A |
| LI_CISPLATIN_RESISTANCE_UP | 3 | 12.77 | 0.001 | THSD7A,TWIST1,ZBTB20 |
| LI_WILMS_TUMOR_VS_FETAL_KIDNEY_2_UP | 3 | 12.77 | 0.001 | ENC1,IGF2,NCAM1 |
| SHI_SPARC_TARGETS_UP | 3 | 11.61 | 0.002 | PIK3R1,PRKCB,SEMA6A |
| HUPER_BREAST_BASAL_VS_LUMINAL_DN | 3 | 6.72 | 0.009 | KRT18,KRT19,MYO5C |
| KEEN_RESPONSE_TO_ROSIGLITAZONE_UP | 3 | 5.81 | 0.014 | DGAT1,NDUFB10,PPARA |
| JOSEPH_RESPONSE_TO_SODIUM_BUTYRATE_DN | 3 | 5.32 | 0.018 | ABCC2,PRKAR2A,THSD7A |
| MARIADASON_REGULATED_BY_HISTONE_ACETYLATION_DN | 3 | 5.11 | 0.020 | MPDU1,PCGF3,YY1 |
| ZHAN_MULTIPLE_MYELOMA_CD1_VS_CD2_DN | 3 | 4.91 | 0.022 | DENND5B,PRDM5,PRKCB |
| SANSOM_WNT_PATHWAY_REQUIRE_MYC | 3 | 4.91 | 0.022 | MTAP,SOX17,TNFRSF19 |
| ZHANG_GATA6_TARGETS_DN | 3 | 4.91 | 0.022 | PCBP3,RAB27A,SREBF2 |
| SOTIRIOU_BREAST_CANCER_GRADE_1_VS_3_DN | 3 | 4.73 | 0.025 | RUNX1,SESN1,TP73-AS1 |
| WESTON_VEGFA_TARGETS_6HR | 3 | 4.73 | 0.025 | FAP,KRT18,SEMA5A |
| RAMALHO_STEMNESS_DN | 3 | 4.56 | 0.027 | AP2A2,NCAM1,PRKCB |
| HUMMERICH_SKIN_CANCER_PROGRESSION_UP | 3 | 4.40 | 0.030 | APC,KRT19,S100A6 |
| ZAMORA_NOS2_TARGETS_UP | 3 | 4.40 | 0.030 | KRT19,RRM1,SMS |
| BROWNE_INTERFERON_RESPONSIVE_GENES | 3 | 4.40 | 0.030 | CCL8,PDZD2,ZBTB20 |
| BAELDE_DIABETIC_NEPHROPATHY_UP | 3 | 4.40 | 0.030 | ARHGEF15,MT1G,SEMA6A |
| OUELLET_CULTURED_OVARIAN_CANCER_INVASIVE_VS_LMP_UP | 3 | 4.26 | 0.032 | FLII,KRT18,KRT19 |
| YAO_HOXA10_TARGETS_VIA_PROGESTERONE_UP | 3 | 4.26 | 0.032 | AKAP8L,NCAM1,PIK3R1 |
| RIZKI_TUMOR_INVASIVENESS_2D_UP | 3 | 4.26 | 0.032 | COL4A6,GNA11,IGF2 |
| LINDVALL_IMMORTALIZED_BY_TERT_UP | 3 | 4.12 | 0.035 | ELOVL5,KRT18,VAMP3 |
| NAKAYAMA_SOFT_TISSUE_TUMORS_PCA1_UP | 3 | 4.12 | 0.035 | FAP,HLA-DPA1,S100A6 |
| DAVICIONI_TARGETS_OF_PAX_FOXO1_FUSIONS_DN | 3 | 3.99 | 0.038 | ADORA1,NEFM,YY1 |
| KUNINGER_IGF1_VS_PDGFB_TARGETS_UP | 3 | 3.99 | 0.038 | HSPB2,IGF2,NCAM1 |
| XU_HGF_TARGETS_REPRESSED_BY_AKT1_DN | 3 | 3.87 | 0.041 | GTDC1,MTAP,NEK1 |
| LEIN_PONS_MARKERS | 3 | 3.87 | 0.041 | ABHD4,ESYT1,MTAP |
| FERRANDO_T_ALL_WITH_MLL_ENL_FUSION_UP | 3 | 3.76 | 0.045 | AKAP1,SREBF2,WAS |
| DUTERTRE_ESTRADIOL_RESPONSE_6HR_DN | 3 | 3.65 | 0.048 | LIMA1,MTAP,SH3BP4 |
| BENPORATH_NANOG_TARGETS | 2 | 0.20 | 0.002 | COL4A6,KRT18 |
| LEI_HOXC8_TARGETS_DN | 2 | 17.03 | 0.005 | KRT19,NCAM1 |
| SCHAEFFER_PROSTATE_DEVELOPMENT_AND_CANCER_BOX4_UP | 2 | 14.19 | 0.008 | KRT19,PRKCB |
| WENG_POR_TARGETS_GLOBAL_DN | 2 | 14.19 | 0.008 | ELOVL5,MTAP |
| MISHRA_CARCINOMA_ASSOCIATED_FIBROBLAST_DN | 2 | 10.64 | 0.014 | KRT18,KRT19 |
| WEST_ADRENOCORTICAL_CARCINOMA_VS_ADENOMA_DN | 2 | 10.64 | 0.014 | DGAT1,HLA-DPA1 |
| DACOSTA_ERCC3_ALLELE_XPCS_VS_TTD_UP | 2 | 9.46 | 0.018 | ADORA1,KRT18 |
| NIELSEN_GIST_VS_SYNOVIAL_SARCOMA_UP | 2 | 9.46 | 0.018 | DLX5,ENC1 |
| GENTILE_UV_RESPONSE_CLUSTER_D8 | 2 | 9.46 | 0.018 | TWIST1,YY1 |
| HINATA_NFKB_TARGETS_KERATINOCYTE_DN | 2 | 9.46 | 0.018 | ESYT1,RUNX1 |
| GARGALOVIC_RESPONSE_TO_OXIDIZED_PHOSPHOLIPIDS_RED_DN | 2 | 8.52 | 0.022 | MAFB,SESN1 |
| AIYAR_COBRA1_TARGETS_DN | 2 | 8.52 | 0.022 | CPD,MYO5C |
| CUI_TCF21_TARGETS_UP | 2 | 8.52 | 0.022 | FAP,NCAM1 |
| MCCABE_HOXC6_TARGETS_CANCER_UP | 2 | 8.52 | 0.022 | HSPB2,RUNX1 |
| HOFMANN_MYELODYSPLASTIC_SYNDROM_LOW_RISK_UP | 2 | 8.52 | 0.022 | PRKCB,RUNX1 |
| GAZDA_DIAMOND_BLACKFAN_ANEMIA_ERYTHROID_UP | 2 | 7.74 | 0.026 | NUFIP1,SESN1 |
| TAKEDA_TARGETS_OF_NUP98_HOXA9_FUSION_3D_DN | 2 | 7.10 | 0.031 | CLEC10A,MAFB |
| YANG_BREAST_CANCER_ESR1_BULK_UP | 2 | 7.10 | 0.031 | ELOVL5,MYO5C |
| JI_METASTASIS_REPRESSED_BY_STK11 | 2 | 7.10 | 0.031 | ID4,LIMCH1 |
| DORSAM_HOXA9_TARGETS_DN | 2 | 7.10 | 0.031 | IGF2,KRT18 |
| MARIADASON_RESPONSE_TO_BUTYRATE_SULINDAC_4 | 2 | 7.10 | 0.031 | APTX,CACNA1D |
| LOPES_METHYLATED_IN_COLON_CANCER_UP | 2 | 7.10 | 0.031 | MT1G,TWIST1 |
| JIANG_TIP30_TARGETS_DN | 2 | 7.10 | 0.031 | AKAP1,IGF2 |
| WIKMAN_ASBESTOS_LUNG_CANCER_DN | 2 | 6.55 | 0.036 | MAFB,RUNX1 |
| RODRIGUES_NTN1_AND_DCC_TARGETS | 2 | 6.55 | 0.036 | PPARA,RAB26 |
| LIANG_HEMATOPOIESIS_STEM_CELL_NUMBER_SMALL_VS_HUGE_DN | 2 | 6.55 | 0.036 | APC,S100A6 |
| CHEN_HOXA5_TARGETS_9HR_DN | 2 | 6.55 | 0.036 | AKAP1,PCDHGC3 |
| GU_PDEF_TARGETS_DN | 2 | 6.55 | 0.036 | KRT18,KRT19 |
| APPIERTO_RESPONSE_TO_FENRETINIDE_UP | 2 | 6.08 | 0.041 | ABHD4,PDZD2 |
| LI_LUNG_CANCER | 2 | 6.08 | 0.041 | KRT18,KRT19 |
| SEITZ_NEOPLASTIC_TRANSFORMATION_BY_8P_DELETION_DN | 2 | 6.08 | 0.041 | LIMCH1,RCN2 |
| PLASARI_TGFB1_SIGNALING_VIA_NFIC_10HR_DN | 2 | 6.08 | 0.041 | HEY1,MAFB |
| GUILLAUMOND_KLF10_TARGETS_DN | 2 | 6.08 | 0.041 | AACS,ELOVL5 |
| MODY_HIPPOCAMPUS_NEONATAL | 2 | 5.68 | 0.047 | NCAM1,ST8SIA2 |
| DORN_ADENOVIRUS_INFECTION_24HR_DN | 2 | 5.68 | 0.047 | KRT19,LIMA1 |
| KIM_ALL_DISORDERS_OLIGODENDROCYTE_NUMBER_CORR_UP | 1 | 0.13 | 0.004 | ESYT1 |
| KIM_ALL_DISORDERS_CALB1_CORR_UP | 1 | 0.18 | 0.022 | RCN2 |
| LIU_CDX2_TARGETS_DN | 1 | 42.58 | 0.023 | KRT19 |
| BERENJENO_TRANSFORMED_BY_RHOA_REVERSIBLY_UP | 1 | 42.58 | 0.023 | PIK3R1 |
| SMID_BREAST_CANCER_NORMAL_LIKE_DN | 1 | 42.58 | 0.023 | KRT18 |
| KAPOSI_LIVER_CANCER_MET_DN | 1 | 42.58 | 0.023 | PIK3R1 |
| WANG_THOC1_TARGETS_UP | 1 | 42.58 | 0.023 | KRT18 |
| MARTINEZ_TP53_TARGETS_DN | 1 | 0.20 | 0.037 | ENC1 |
| IVANOVA_HEMATOPOIESIS_LATE_PROGENITOR | 1 | 0.21 | 0.041 | CLEC10A |
| MYLLYKANGAS_AMPLIFICATION_HOT_SPOT_27 | 1 | 21.29 | 0.046 | APC |
| MYLLYKANGAS_AMPLIFICATION_HOT_SPOT_1 | 1 | 21.29 | 0.046 | WAS |
| MCGOWAN_RSP6_TARGETS_DN | 1 | 21.29 | 0.046 | ZNF707 |
| DONATO_CELL_CYCLE_TRETINOIN | 1 | 21.29 | 0.046 | ABI1 |
| TSUDA_ALVEOLAR_SOFT_PART_SARCOMA | 1 | 21.29 | 0.046 | PRKAG2 |
| MIKKELSEN_DEDIFFERENTIATED_STATE_UP | 1 | 21.29 | 0.046 | DSG2 |
| TCGA_GLIOBLASTOMA_MUTATED | 1 | 21.29 | 0.046 | PIK3R1 |
| RAFFEL_VEGFA_TARGETS_DN | 1 | 21.29 | 0.046 | HEY1 |
