# Supplementary material for: Clinical relevance of the transcriptional signature regulated by CDC42 in colorectal cancer
Source: Oncotarget. 2017 Mar 1;8(16):26755–70. doi: 10.18632/oncotarget.15815 (PMC5432295; doi:10.18632/oncotarget.15815)
Supplement: Supplementary file 4 [file oncotarget-08-26755-s004.docx]

**Table S4: 57 genes correlated with CDC42 transcriptional signature in TCGA CRC patients** (red: up-regulated genes, blue: down-regulated genes)

| Ensembl Gene ID | hgnc_symbol | Log2FoldChange | adj.p.value |
| --- | --- | --- | --- |
| ENSG00000025800 | KPNA6 | 0.372 | 5.1E-19 |
| ENSG00000038295 | TLL1 | 0.428 | 4.8E-03 |
| ENSG00000049245 | VAMP3 | 0.556 | 6.3E-45 |
| ENSG00000064042 | LIMCH1 | 0.632 | 5.5E-09 |
| ENSG00000069974 | RAB27A | 0.590 | 2.1E-19 |
| ENSG00000102172 | SMS | 0.418 | 2.9E-15 |
| ENSG00000108582 | CPD | 0.269 | 6.5E-05 |
| ENSG00000108700 | CCL8 | 0.828 | 4.2E-07 |
| ENSG00000109787 | KLF3 | 0.352 | 1.4E-14 |
| ENSG00000115084 | SLC35F5 | 0.413 | 3.3E-11 |
| ENSG00000117676 | RPS6KA1 | 0.278 | 5.3E-06 |
| ENSG00000128833 | MYO5C | 0.228 | 1.4E-03 |
| ENSG00000129255 | MPDU1 | 0.224 | 6.6E-04 |
| ENSG00000132623 | ANKEF1 | 0.364 | 3.6E-07 |
| ENSG00000137145 | DENND4C | 0.192 | 7.1E-03 |
| ENSG00000137601 | NEK1 | 0.461 | 4.5E-12 |
| ENSG00000148153 | INIP | 0.193 | 5.5E-07 |
| ENSG00000151789 | ZNF385D | 0.344 | 2.6E-02 |
| ENSG00000163293 | NIPAL1 | 0.554 | 1.9E-14 |
| ENSG00000166501 | PRKCB | 0.437 | 1.7E-04 |
| ENSG00000167325 | RRM1 | 0.272 | 2.0E-08 |
| ENSG00000170456 | DENND5B | 0.349 | 1.4E-04 |
| ENSG00000177853 | ZNF518A | 0.448 | 9.1E-09 |
| ENSG00000197956 | S100A6 | 0.477 | 3.4E-09 |
| ENSG00000204787 | REG1CP | 0.714 | 3.3E-02 |
| ENSG00000213593 | TMX2 | 0.250 | 8.2E-08 |
| ENSG00000242808 | SOX2-OT | 0.649 | 2.1E-04 |
| ENSG00000247556 | OIP5-AS1 | 0.331 | 1.1E-08 |
| ENSG00000264229 | RNU4ATAC | 0.830 | 3.2E-05 |
| ENSG00000275718 | CCL15 | 0.596 | 3.8E-10 |
| ENSG00000007402 | CACNA2D2 | -0.657 | 1.4E-05 |
| ENSG00000011243 | AKAP8L | -0.425 | 1.1E-24 |
| ENSG00000011376 | LARS2 | -0.223 | 2.8E-05 |
| ENSG00000033011 | ALG1 | -0.182 | 2.2E-04 |
| ENSG00000081760 | AACS | -0.216 | 1.7E-04 |
| ENSG00000085511 | MAP3K4 | -0.152 | 1.0E-04 |
| ENSG00000100403 | ZC3H7B | -0.330 | 2.2E-09 |
| ENSG00000105880 | DLX5 | -0.705 | 3.6E-04 |
| ENSG00000108306 | FBXL20 | -0.135 | 5.9E-03 |
| ENSG00000112640 | PPP2R5D | -0.127 | 5.6E-03 |
| ENSG00000113761 | ZNF346 | -0.144 | 2.3E-04 |
| ENSG00000132394 | EEFSEC | -0.514 | 1.0E-21 |
| ENSG00000134452 | FBXO18 | -0.170 | 2.8E-07 |
| ENSG00000140990 | NDUFB10 | -0.182 | 1.6E-02 |
| ENSG00000141556 | TBCD | -0.536 | 1.1E-19 |
| ENSG00000142330 | CAPN10 | -0.544 | 2.0E-28 |
| ENSG00000157388 | CACNA1D | -0.542 | 3.0E-08 |
| ENSG00000158352 | SHROOM4 | -0.401 | 5.8E-05 |
| ENSG00000160200 | CBS | -1.054 | 3.3E-08 |
| ENSG00000161010 | C5orf45 | -0.574 | 3.3E-21 |
| ENSG00000181135 | ZNF707 | -0.557 | 4.8E-20 |
| ENSG00000183570 | PCBP3 | -0.341 | 9.3E-03 |
| ENSG00000197565 | COL4A6 | -0.327 | 4.8E-02 |
| ENSG00000198911 | SREBF2 | -0.386 | 7.1E-11 |
| ENSG00000240184 | PCDHGC3 | -0.366 | 2.2E-03 |
| ENSG00000260233 | SSSCA1-AS1 | -0.617 | 1.0E-21 |
| ENSG00000274276 | CBSL | -1.038 | 2.8E-05 |
