# Supplementary material for: Clinical relevance of the transcriptional signature regulated by CDC42 in colorectal cancer
Source: Oncotarget. 2017 Mar 1;8(16):26755–70. doi: 10.18632/oncotarget.15815 (PMC5432295; doi:10.18632/oncotarget.15815)
Supplement: Supplementary file 5 [file oncotarget-08-26755-s005.docx]

**Table S5: Full table of Gene set enrichment analyses for curated gene sets/ canonical pathways (p-value < 0.05) for CDC42 regulated genes in TCGA datasets**

| Term | Count | Fold enrichment | PValue | Genes |
| --- | --- | --- | --- | --- |
| WAKABAYASHI_ADIPOGENESIS_PPARG_BOUND_8D | 6 | 2.98 | 0.014 | AACS,DLX5,FBXO18,MPDU1,NDUFB10,PPP2R5D |
| CREIGHTON_ENDOCRINE_THERAPY_RESISTANCE_3 | 6 | 2.55 | 0.029 | ALG1,CPD,LIMCH1,RAB27A,RPS6KA1,S100A6 |
| WONG_ADULT_TISSUE_STEM_MODULE | 6 | 2.52 | 0.030 | CACNA2D2,LIMCH1,MYO5C,PRKCB,RAB27A,S100A6 |
| MARTINEZ_TP53_TARGETS_UP | 5 | 2.55 | 0.045 | CPD,KLF3,KPNA6,RAB27A,RRM1 |
| MULLIGHAN_MLL_SIGNATURE_1_UP | 4 | 3.25 | 0.034 | RAB27A,RPS6KA1,S100A6,TBCD |
| ZHANG_GATA6_TARGETS_DN | 3 | 14.74 | 0.001 | PCBP3,RAB27A,SREBF2 |
| BOYAULT_LIVER_CANCER_SUBCLASS_G1_UP | 3 | 8.15 | 0.006 | CACNA1D,MYO5C,SREBF2 |
| HOSHIDA_LIVER_CANCER_SUBCLASS_S2 | 3 | 8.15 | 0.006 | CPD,KLF3,SREBF2 |
| CHIARADONNA_NEOPLASTIC_TRANSFORMATION_KRAS_UP | 3 | 7.66 | 0.007 | PRKCB,RRM1,SMS |
| PANGAS_TUMOR_SUPPRESSION_BY_SMAD1_AND_SMAD5_DN | 3 | 6.84 | 0.009 | CBS,SREBF2,TMX2 |
| JIANG_VHL_TARGETS | 3 | 5.90 | 0.014 | FBXO18,PPP2R5D,RPS6KA1 |
| CAIRO_HEPATOBLASTOMA_UP | 3 | 4.56 | 0.028 | CPD,MAP3K4,SREBF2 |
| BROWNE_HCMV_INFECTION_20HR_UP | 3 | 4.03 | 0.038 | AKAP8L,KPNA6,MPDU1 |
| WAMUNYOKOLI_OVARIAN_CANCER_LMP_UP | 3 | 3.99 | 0.039 | AACS,RPS6KA1,S100A6 |
| AIYAR_COBRA1_TARGETS_DN | 2 | 25.55 | 0.003 | CPD,MYO5C |
| YU_MYC_TARGETS_DN | 2 | 12.77 | 0.010 | KLF3,PRKCB |
| SONG_TARGETS_OF_IE86_CMV_PROTEIN | 2 | 12.77 | 0.010 | RAB27A,RRM1 |
| ZHAN_MULTIPLE_MYELOMA_CD1_VS_CD2_DN | 2 | 9.83 | 0.017 | DENND5B,PRKCB |
| KIM_GERMINAL_CENTER_T_HELPER_UP | 2 | 9.12 | 0.020 | SLC35F5,ZNF518A |
| ZAMORA_NOS2_TARGETS_UP | 2 | 8.81 | 0.021 | RRM1,SMS |
| VERNELL_RETINOBLASTOMA_PATHWAY_UP | 2 | 7.98 | 0.026 | RAB27A,RRM1 |
| WATTEL_AUTONOMOUS_THYROID_ADENOMA_UP | 2 | 7.51 | 0.029 | CACNA1D,PRKCB |
| LABBE_WNT3A_TARGETS_DN | 2 | 6.90 | 0.034 | PRKCB,S100A6 |
| GRABARCZYK_BCL11B_TARGETS_UP | 2 | 6.72 | 0.035 | RAB27A,SLC35F5 |
| HOEBEKE_LYMPHOID_STEM_CELL_DN | 2 | 6.55 | 0.037 | LIMCH1,S100A6 |
| ROSS_AML_WITH_AML1_ETO_FUSION | 2 | 6.55 | 0.037 | CACNA2D2,RPS6KA1 |
| LINDSTEDT_DENDRITIC_CELL_MATURATION_A | 2 | 6.55 | 0.037 | CCL15,CCL8 |
| IGARASHI_ATF4_TARGETS_DN | 2 | 6.39 | 0.039 | CBS,DENND5B |
| FLECHNER_BIOPSY_KIDNEY_TRANSPLANT_REJECTED_VS_OK_UP | 2 | 6.39 | 0.039 | PRKCB,RAB27A |
| NELSON_RESPONSE_TO_ANDROGEN_UP | 2 | 6.39 | 0.039 | CPD,SMS |
| MAHAJAN_RESPONSE_TO_IL1A_UP | 2 | 6.39 | 0.039 | AKAP8L,CCL8 |
| HOEBEKE_LYMPHOID_STEM_CELL_UP | 2 | 6.08 | 0.042 | PRKCB,TBCD |
| CHEN_LIVER_METABOLISM_QTL_CIS | 2 | 5.94 | 0.044 | CAPN10,SLC35F5 |
| KOBAYASHI_EGFR_SIGNALING_24HR_UP | 2 | 5.81 | 0.046 | CPD,LIMCH1 |
| GRAHAM_CML_DIVIDING_VS_NORMAL_QUIESCENT_DN | 2 | 5.68 | 0.048 | LIMCH1,MYO5C |
| KANG_IMMORTALIZED_BY_TERT_UP | 2 | 5.68 | 0.048 | COL4A6,LARS2 |
| NIKOLSKY_BREAST_CANCER_17Q11_Q21_AMPLICON | 2 | 5.55 | 0.050 | CPD,FBXL20 |
| GRADE_COLON_AND_RECTAL_CANCER_DN | 2 | 5.55 | 0.050 | CACNA2D2,CBS |
| MCGOWAN_RSP6_TARGETS_DN | 1 | 63.86 | 0.016 | ZNF707 |
| OZANNE_AP1_TARGETS_DN | 1 | 42.58 | 0.023 | PCDHGC3 |
| HESSON_TUMOR_SUPPRESSOR_CLUSTER_3P21_3 | 1 | 42.58 | 0.023 | CACNA2D2 |
| KANG_GIST_WITH_PDGFRA_DN | 1 | 42.58 | 0.023 | RPS6KA1 |
| SHARMA_PILOCYTIC_ASTROCYTOMA_LOCATION_DN | 1 | 42.58 | 0.023 | CBS |
| WATANABE_ULCERATIVE_COLITIS_WITH_CANCER_UP | 1 | 31.93 | 0.031 | LIMCH1 |
| PETRETTO_BLOOD_PRESSURE_UP | 1 | 31.93 | 0.031 | KPNA6 |
| NIKOLSKY_BREAST_CANCER_21Q22_AMPLICON | 1 | 31.93 | 0.031 | PCBP3 |
| SCHURINGA_STAT5A_TARGETS_UP | 1 | 31.93 | 0.031 | ZNF518A |
| ZAIDI_OSTEOBLAST_TRANSCRIPTION_FACTORS | 1 | 31.93 | 0.031 | DLX5 |
| MARKS_ACETYLATED_NON_HISTONE_PROTEINS | 1 | 25.55 | 0.039 | KPNA6 |
| PETRETTO_LEFT_VENTRICLE_MASS_QTL_CIS_DN | 1 | 25.55 | 0.039 | ZNF346 |
| LAMB_CCND1_TARGETS | 1 | 25.55 | 0.039 | RPS6KA1 |
| HOWLIN_CITED1_TARGETS_2_DN | 1 | 25.55 | 0.039 | VAMP3 |
| YANAGISAWA_LUNG_CANCER_RECURRENCE | 1 | 25.55 | 0.039 | S100A6 |
| WILLIAMS_ESR2_TARGETS_DN | 1 | 21.29 | 0.046 | S100A6 |
| SCHAEFFER_PROSTATE_DEVELOPMENT_AND_CANCER_BOX4_UP | 1 | 21.29 | 0.046 | PRKCB |
| LI_CYTIDINE_ANALOG_PATHWAY | 1 | 21.29 | 0.046 | RRM1 |
| FINETTI_BREAST_CANCER_KINOME_GREEN | 1 | 21.29 | 0.046 | PRKCB |
| MEISSNER_BRAIN_HCP_WITH_H3K4ME2 | 1 | 21.29 | 0.046 | MYO5C |
| KAMIKUBO_MYELOID_MN1_NETWORK | 1 | 21.29 | 0.046 | RAB27A |
